# MicroRNAs in Cardiovascular Diseases: Molecular Networks of Cellular Homeostasis, Inflammation, and Pathological Remodeling

**DOI:** 10.3390/ijms27083582

**Published:** 2026-04-17

**Authors:** Humberto Vélez-Slimani, Luis A. Salazar

**Affiliations:** 1 Doctoral Program in Sciences Major in Applied Cellular and Molecular Biology, Universidad de La Frontera, Temuco 4811230, Chile; h.velez01@ufromail.cl; 2 Center for Molecular Biology and Pharmacogenetics, Department of Basic Sciences, Universidad de La Frontera, Temuco 4811230, Chile

**Keywords:** microRNAs, cardiovascular diseases, inflammation, fibrosis, vascular remodeling, biomarkers, extracellular vesicles, RNA therapeutics

## Abstract

Cardiovascular diseases remain the leading cause of morbidity and mortality worldwide, underscoring the need to better define the molecular mechanisms that govern cardiovascular homeostasis and disease progression. Among post-transcriptional regulators, microRNAs have emerged as important modulators of endothelial function, vascular smooth muscle cell plasticity, cardiomyocyte integrity, and cardiac fibroblast activity. This narrative review examines how microRNAs orchestrate molecular networks linking cellular homeostasis to inflammation, oxidative stress, mitochondrial dysfunction, apoptosis, fibrosis, angiogenesis, and pathological remodeling across major cardiovascular cell types. It further discusses how these regulatory programs are reflected in specific cardiovascular diseases, including atherosclerosis, hypertension, acute myocardial infarction, heart failure, and arrhythmias. In addition, the review addresses the growing relevance of circulating and extracellular vesicle-associated microRNAs as candidate biomarkers for diagnosis, prognosis, and disease monitoring, as well as their therapeutic potential through mimics, inhibitors, antagomirs, and emerging delivery systems. Finally, current translation barriers are considered, including methodological heterogeneity, limited tissue specificity, delivery challenges, safety concerns, and the need for large-scale clinical validation. Overall, microRNAs are presented as integrative regulators connecting cardiovascular cell biology with disease mechanisms and clinical applications.

## 1. Introduction

Cardiovascular diseases remain the leading cause of death worldwide and impose a disproportionate health burden, particularly in low and middle-income countries. The scale of this problem, together with the persistence of premature events and the major contribution of myocardial infarction and stroke to global mortality, underscores the need to further elucidate the molecular mechanisms that govern the onset and progression of cardiovascular damage [1].

Among post-transcriptional regulators, microRNAs have emerged as fine-tuners of gene expression, capable of modulating the stability and translation of multiple target mRNAs. Although they have classically been described as components of RISC-mediated gene silencing, it is now recognized that their biogenesis, subcellular localization, and regulatory functions are more complex, thereby broadening their biological impact under both physiological and pathological conditions [2,3]. Within the cardiovascular system, microRNAs actively participate in the preservation of cellular homeostasis by regulating essential processes in cardiomyocytes, endothelial cells, vascular smooth muscle cells, and cardiac fibroblasts. Through these actions, they modulate pathways linked to inflammation, oxidative stress, lipid metabolism, endothelial function, apoptosis, and tissue remodeling; consequently, their dysregulation may contribute to both vascular injury and the progression of pathological cardiac remodeling [4,5].

This narrative review aims to provide a focused and critical synthesis of how microRNAs regulate cardiovascular homeostasis, inflammation, and pathological remodeling. Rather than attempting an exhaustive systematic compilation of all available studies, it integrates representative and influential evidence across major cardiovascular cell types, shared pathogenic mechanisms, disease-specific settings, biomarker research, and emerging therapeutic strategies. The rationale for undertaking this review is that, although multiple reviews on cardiovascular microRNAs are already available, many focus predominantly on individual diseases, translational applications, or selected mechanistic pathways. Here, we sought to provide a broader integrative framework linking cell-type-specific homeostatic roles, shared pathological networks, clinical manifestations, and translational perspectives within a single narrative structure. Particular emphasis is placed on translational relevance, methodological heterogeneity, and areas in which the current evidence remains limited, inconsistent, or insufficiently validated. This framework is intended to position microRNAs not as isolated regulators, but as integrative nodes linking cardiovascular biology to clinical application.

## 2. Materials and Methods

This article was designed as a narrative review rather than a systematic review or meta-analysis. A structured literature search was conducted in PubMed/MEDLINE, Scopus, and Web of Science to identify relevant studies addressing the role of microRNAs in cardiovascular biology and disease. Search terms combined keywords such as “microRNA”, “miRNA”, “cardiovascular disease”, “atherosclerosis”, “hypertension”, “myocardial infarction”, “heart failure”, “arrhythmia”, “biomarker”, “extracellular vesicle”, and “therapy”. Additional relevant publications were identified through citation tracking of key articles and recent reviews.

Studies were considered for inclusion if they addressed mechanistic, diagnostic, prognostic, or therapeutic aspects of microRNAs in cardiovascular systems or cardiovascular diseases. Priority was given to peer-reviewed original studies, landmark reports, recent reviews, and articles with translational relevance, mechanistic validation, or human clinical data. In cases of overlapping literature, preference was given to more recent, comprehensive, or methodologically informative publications. Conference abstracts, non-peer-reviewed sources, and studies not directly related to cardiovascular microRNAs were generally excluded. Particular attention was given to studies reporting analytical platform, biospecimen type, cohort characteristics, sample size, and, when available, independent validation or replication across populations.

The selected literature was synthesized narratively and organized into major thematic domains, including cardiovascular homeostasis, microRNA-regulated pathological networks, specific cardiovascular diseases, biomarker development, therapeutic strategies, and current translational challenges. Because the aim of this review was conceptual integration rather than exhaustive systematic coverage, the evidence was interpreted critically with attention to biological context, methodological heterogeneity, translational relevance, and current limitations of the field. This approach was selected to support an integrative and hypothesis-oriented discussion of cardiovascular microRNAs across biological levels and disease settings, rather than to produce a disease-restricted or purely catalog-based summary.

Because this article was designed as a narrative review rather than a systematic review or meta-analysis, no formal risk-of-bias tool was applied across all included studies. Nevertheless, the literature was critically appraised using qualitative criteria relevant to translational cardiovascular microRNA research, including study design, experimental model, sample size, biospecimen definition, mechanistic validation, replication across independent cohorts or populations, and clinical versus preclinical relevance. In general, evidence derived from multicohort human studies, independent validation datasets, or convergent human and mechanistic experimental findings was considered stronger than evidence based on small exploratory cohorts, single-center studies, or isolated preclinical observations. A qualitative summary of representative studies included in this review, together with their study design, validation features, key limitations, and qualitative strength of evidence, is provided in Supplementary Table S1.

## 3. MicroRNAs in Cardiovascular Homeostasis

MicroRNAs are integral components of cardiovascular homeostasis because they coordinate post-transcriptional regulatory programs across the major cellular compartments of the cardiovascular system, including endothelial cells, vascular smooth muscle cells, cardiomyocytes, and cardiac fibroblasts. Rather than acting within isolated pathways, these small non-coding RNAs organize cell-type-specific molecular networks that govern vascular tone, redox balance, angiogenesis, contractile function, extracellular matrix turnover, and intercellular communication. Consequently, perturbations in microRNA expression may shift adaptive physiological responses toward inflammation, fibrosis, and maladaptive remodeling, thereby linking normal cardiovascular maintenance with the earliest stages of disease development [5,6].

### 3.1. Vascular Endothelium

The vascular endothelium is a dynamic monolayer that plays a central role in cardiovascular homeostasis by regulating vascular tone, permeability, leukocyte trafficking, thrombosis, and angiogenesis. Within this context, microRNAs have emerged as essential post-transcriptional regulators of endothelial identity and function, fine-tuning the expression of genes involved in nitric oxide bioavailability, inflammatory activation, redox balance, and vascular repair [7,8].

A growing body of evidence indicates that endothelial microRNAs are not merely passive modulators, but integral components of vascular adaptation to physiological and pathological stimuli. Experimental disruption of microRNA processing in endothelial cells has demonstrated that these molecules are required for proper endothelial proliferation, migration, and angiogenic responses, supporting the concept that microRNA-dependent regulation is indispensable for the maintenance of vascular homeostasis [8,9]. Among endothelial-enriched microRNAs, miR-126 is considered one of the most representative examples of vasculoprotective regulation. This microRNA has been associated with the preservation of vascular integrity, the modulation of VEGF signaling, and the control of endothelial inflammatory activation. In addition, evidence indicates that miR-126 limits leukocyte adhesion by repressing vascular cell adhesion molecule 1 (VCAM-1) expression, thereby linking endothelial microRNA regulation to both vascular stability and anti-inflammatory defense [9,10,11].

Beyond individual microRNAs, endothelial biology is also shaped by flow-sensitive and stress-responsive microRNA programs. These regulatory networks respond to hemodynamic cues such as laminar or disturbed shear stress and contribute to the endothelial phenotypes associated with vascular protection or dysfunction. Consequently, endothelial microRNAs are now regarded as molecular nodes connecting biomechanical stimuli with inflammation, atherogenesis, and vascular remodeling [12].

Overall, endothelial microRNAs help integrate angiogenic signaling, inflammatory restraint, and vascular adaptation, and their dysregulation is increasingly linked to early endothelial dysfunction and atherosclerotic progression [13,14].

### 3.2. Vascular Smooth Muscle Cells

Vascular smooth muscle cells (VSMCs) are essential components of the arterial wall and contribute directly to vascular tone, mechanical support, and vessel integrity. Under physiological conditions, they predominantly maintain a contractile phenotype; however, they also retain a remarkable degree of plasticity that allows them to shift toward synthetic, proliferative, migratory, and matrix-remodeling states in response to environmental cues. Although this adaptive flexibility is necessary for vascular repair, its persistent dysregulation is now recognized as a major determinant of pathological vascular remodeling [15,16].

MicroRNAs are central regulators of this phenotypic equilibrium because they fine-tune gene networks involved in differentiation, cytoskeletal organization, cell-cycle progression, apoptosis, and migratory behavior. Rather than acting on isolated targets, they coordinate broader post-transcriptional programs that determine whether VSMCs preserve homeostatic functions or acquire disease-associated features. For this reason, microRNA dysregulation is increasingly viewed as a mechanistically relevant event linking vascular adaptation to vascular pathology [17]. Among the best-characterized examples, the miR-143/145 cluster has been identified as a pivotal determinant of VSMC identity. These microRNAs promote smooth muscle differentiation, support the contractile phenotype, and repress proliferative reprogramming by targeting transcriptional networks involved in phenotype switching. Their downregulation in injured or atherosclerotic vessels further supports the concept that loss of miR-143/145 contributes to the transition from vascular homeostasis to pathological remodeling [18]. By contrast, other microRNAs are more closely associated with the synthetic and proliferative VSMC phenotype. In particular, miR-21 and miR-221/222 have been repeatedly linked to enhanced proliferation, migration, and neointimal growth in response to vascular injury, highlighting that specific microRNA signatures may actively drive remodeling rather than merely reflect it. This illustrates the context-dependent nature of microRNA signaling in VSMCs, where distinct regulatory profiles may either preserve vascular stability or promote lesion progression [19,20].

Overall, current evidence indicates that microRNAs are important regulators of VSMC homeostasis, integrating contractility, plasticity, stress responses, and remodeling capacity into tightly controlled molecular programs. Their therapeutic relevance is therefore increasingly recognized, particularly in proliferative vascular disorders in which restoring a more stable VSMC phenotype may represent a meaningful strategy to limit disease progression [21].

### 3.3. Cardiomyocytes

Cardiomyocytes are the principal contractile cells of the heart and are therefore central to the maintenance of cardiac output, rhythmic electrical activity, and myocardial structural integrity. In these cells, microRNAs act as key post-transcriptional regulators of gene networks involved in sarcomere organization, calcium handling, electrophysiological stability, metabolic adaptation, and stress responsiveness. As a result, cardiomyocyte homeostasis depends not only on transcriptional control but also on a finely balanced microRNA landscape that preserves functional specialization under basal conditions [22]. The importance of microRNAs for cardiomyocyte maintenance is strongly supported by loss-of-function models targeting the microRNA-processing machinery. Cardiac-specific deletion of Dicer, an essential enzyme for microRNA maturation, results in rapidly progressive dilated cardiomyopathy, heart failure, postnatal lethality, misexpression of contractile proteins, and marked sarcomeric disarray. These observations established that intact microRNA biogenesis is indispensable for preserving cardiomyocyte structure and function [23].

Within this regulatory framework, cardiac-enriched microRNAs such as miR-1, miR-133, and the miR-208 family, particularly miR-208a, have been particularly associated with the control of cardiomyocyte identity and performance. These myomiRs contribute to the regulation of differentiation status, contractile gene expression, electrical conduction, and adaptive responses to stress, thereby functioning as molecular organizers of cardiac muscle homeostasis. Their relevance lies not in isolated target repression, but in their capacity to coordinate interconnected regulatory programs across multiple cardiomyocyte pathways [24,25]. Among these regulators, miR-1 is especially relevant because it has been closely linked to excitation–contraction coupling and electrophysiological balance. Recent evidence indicates that miR-1 deficiency disrupts cardiac contractility and electrophysiological homeostasis, leading to reduced ejection fraction, impaired repolarization, altered ion channel activity, and increased arrhythmia susceptibility. These findings reinforce the concept that appropriate microRNA dosage is critical for cardiomyocyte function, and that even partial imbalance may have direct pathogenic consequences [26]. Taken together, microRNAs contribute to the coordinated control of structural maintenance, contractile performance, electrophysiological balance, and stress adaptation in cardiomyocytes. Their dysregulation is increasingly associated with maladaptive remodeling, electrical instability, and functional decline [27].

### 3.4. Cardiac Fibroblasts

Cardiac fibroblasts are essential for myocardial homeostasis because they preserve extracellular matrix (ECM) integrity, provide structural support, and participate in paracrine signaling that influences cardiomyocyte maturation, survival, and tissue repair. Recent evidence has further shown that cardiac fibroblasts are not a uniform population, but rather a heterogeneous and dynamic cell compartment whose phenotypic diversity contributes to both physiological adaptation and pathological remodeling [28,29].

Within this framework, microRNAs act as key post-transcriptional regulators of fibroblast identity and behavior by modulating proliferation, migration, phenotypic transition, inflammatory responsiveness, and ECM production. Their biological relevance in fibroblasts extends beyond fibrosis alone, as they help define whether these cells remain in a homeostatic reparative state or undergo maladaptive activation into matrix-producing myofibroblasts. Accordingly, microRNA-dependent regulation is increasingly regarded as a major layer of control over fibroblast plasticity and cardiac tissue remodeling [30].

Among the best-characterized antifibrotic regulators, the miR-29 family has emerged as a canonical suppressor of profibrotic gene expression in cardiac fibroblasts. Classical experimental evidence demonstrated that miR-29 targets multiple ECM-related transcripts, including collagens, fibrillins, and elastin, and that its downregulation after myocardial infarction promotes a profibrotic gene program, whereas its overexpression in fibroblasts reduces collagen expression. These findings established miR-29 as a central molecular brake on excessive matrix deposition [31]. By contrast, miR-21 is widely recognized as a prototypical profibrotic microRNA in the heart. Recent analyses indicate that miR-21 contributes to fibroblast activation and cardiac fibrosis through its interactions with signaling pathways such as TGF-β, ERK, PI3K-Akt, PTEN, and inflammatory mediators, reinforcing the concept that distinct microRNA profiles can differentially shape fibroblast behavior. This opposition between antifibrotic and profibrotic microRNA programs highlights the central role of fibroblast-associated microRNAs in determining whether cardiac remodeling remains adaptive or progresses toward persistent fibrosis [32].

Collectively, microRNAs are increasingly recognized as relevant regulators of fibroblast plasticity, matrix turnover, and maladaptive remodeling in the heart. Their dysregulation is therefore linked not only to fibrotic activation, but also to broader progression of cardiovascular disease [33]. Representative microRNAs involved in cardiovascular cellular homeostasis, together with their principal biological functions, representative targets/pathways, and consequences of dysregulation, are summarized in Table 1.

**Figure 1:**
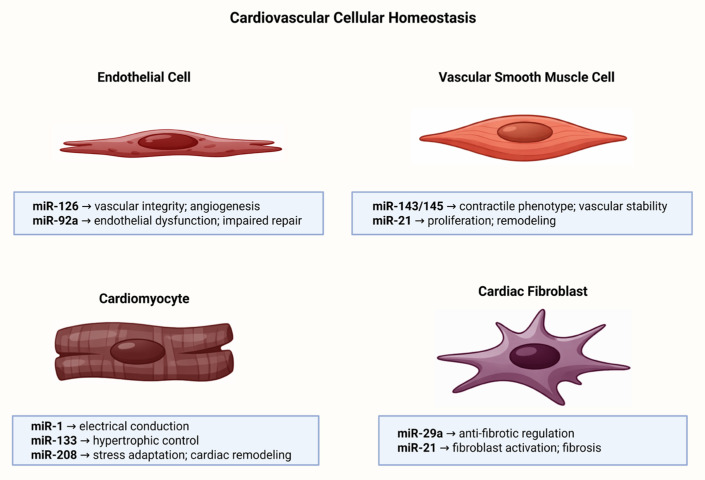
Representative microRNAs involved in cardiovascular cellular homeostasis across major cardiovascular cell types. The scheme summarizes four major cardiovascular cell types—endothelial cells, vascular smooth muscle cells, cardiomyocytes, and cardiac fibroblasts—together with selected microRNAs associated with vascular integrity, contractile phenotype maintenance, electrophysiological balance, cardiac remodeling, and fibroblast activation. Created in BioRender. Vélez Slimani, H. (2026) https://BioRender.com/ttumdtp (accessed on 14 January 2026).

Figure 1 illustrates the major cardiovascular cell types involved in microRNA-mediated homeostasis, together with representative microRNAs and their principal biological functions.

## 4. MicroRNA-Regulated Molecular Networks in Cardiovascular Pathology

The transition from cardiovascular homeostasis to overt disease is driven by the progressive disruption of interconnected molecular networks rather than by isolated cellular defects. In this context, microRNAs have emerged as key integrators of pathological signaling because they coordinate post-transcriptional programs involved in inflammation, oxidative stress, apoptosis, fibrosis, and vascular or myocardial remodeling across multiple cardiovascular cell types. Their regulatory influence is particularly relevant in disease settings, where altered microRNA expression may amplify maladaptive responses, sustain chronic tissue injury, and promote the progression from early molecular imbalance to clinically evident cardiovascular disorders [44,45,46].

### 4.1. Inflammation

Inflammation is now recognized as a central driver of cardiovascular pathology rather than a secondary bystander response. Across vascular and myocardial disease, sterile danger signals, tissue injury, and metabolic stress activate inflammatory cascades that promote endothelial activation, leukocyte recruitment, cytokine release, and progressive tissue damage. Within this setting, microRNAs have emerged as important post-transcriptional regulators of inflammatory signaling, linking innate immune activation to the onset and progression of cardiovascular disorders [45,47].

A particularly relevant concept in this field is that of inflammatory microRNAs, or inflamma-miRs, a subset of microRNAs that modulate cardiovascular inflammation through pathways related to thromboinflammation, atherogenesis, and immune-cell activation. Among the most frequently implicated molecules are miR-21, miR-33, miR-34a, miR-146a, miR-155, and miR-223, which collectively influence inflammatory tone by regulating macrophage behavior, endothelial responses, cytokine production, and signaling downstream of TLR- and NF-κB-related pathways [45].

Among these regulators, miR-146a is generally regarded as a negative-feedback modulator of inflammation because it restrains TLR/IL-1R signaling by targeting mediators such as IRAK1 and TRAF6, thereby limiting NF-κB-driven cytokine production. By contrast, miR-155 is more consistently associated with pro-inflammatory activation, particularly in macrophage-rich and atherosclerotic settings, where it enhances inflammatory signaling and contributes to plaque destabilization. miR-223, in turn, has been linked to myeloid-cell homeostasis and anti-inflammatory restraint, including suppression of endothelial injury and modulation of inflammasome-related signaling. These examples illustrate that inflammatory microRNAs do not act uniformly; instead, they shape cardiovascular inflammation through context-dependent and cell-specific regulatory programs [45,48].

Beyond sterile cardiovascular inflammation, dysregulated microRNA networks are also increasingly relevant in immune-mediated conditions with cardiac involvement. In autoimmune diseases, microRNAs participate in the regulation of innate and adaptive immune responses, immune-cell development, and tolerance-related pathways, supporting the idea that post-transcriptional immune dysregulation may also contribute to cardiovascular injury in immune-mediated settings [49,50]. This concept is particularly evident in autoimmune heart disease, where immune-mediated aggression against cardiac tissue represents a clinically relevant manifestation of cardiovascular inflammation [51]. Likewise, in myocarditis, altered microRNA and broader non-coding RNA signatures have been linked to disease mechanisms and are being explored as potential biomarkers and therapeutic targets; recent reviews further indicate that these regulatory networks are relevant in myocarditis across infectious, autoimmune, and other immune-mediated contexts [52,53].

From a mechanistic and translational perspective, the importance of these molecules lies in their ability to connect inflammatory signaling with endothelial dysfunction, fibrotic remodeling, plaque vulnerability, and adverse myocardial remodeling. Accordingly, inflammatory microRNA signatures are increasingly being viewed not only as mechanistic interfaces between immune activation and cardiovascular disease progression, but also as promising candidates for biomarker development, patient stratification, and future miRNA-based anti-inflammatory interventions [54,55].

### 4.2. Oxidative Stress and Mitochondrial Dysfunction

Oxidative stress and mitochondrial dysfunction are tightly interconnected processes that play a central role in the initiation and progression of cardiovascular disease. In the diseased cardiovascular system, excessive production of reactive oxygen species (ROS), impaired antioxidant defenses, and defective mitochondrial quality control disrupt cellular bioenergetics and damage proteins, lipids, and nucleic acids. These alterations are further amplified by changes in mitochondrial DNA integrity, oxidative phosphorylation, fusion–fission balance, and mitophagy, thereby promoting endothelial dysfunction, myocardial injury, and adverse cardiovascular remodeling [56,57].

MicroRNAs have emerged as important regulators of this redox–mitochondrial axis because they fine-tune signaling pathways involved in ROS generation, antioxidant responses, mitochondrial biogenesis, calcium handling, and cell survival. In addition to regulating cytosolic targets, accumulating evidence supports the existence of mitochondria-associated microRNAs, or mitomiRs, which can directly influence mitochondrial metabolism and stress responses. This regulatory layer is particularly relevant in cardiovascular disease, where microRNA-dependent disturbances in mitochondrial homeostasis may shift adaptive stress responses toward sustained oxidative injury and energetic failure [46,58].

Among the best-studied examples, miR-210 has been shown to exert cardioprotective effects in ischemia–reperfusion settings by modulating mitochondrial bioenergetics and limiting ROS flux, thereby reducing infarct size and preserving cardiac function. By contrast, miR-181c has been linked to maladaptive mitochondrial signaling in the heart, where it promotes mitochondrial calcium uptake and contributes to oxidative stress and injury. These findings illustrate that microRNA-mediated control of mitochondrial function is highly context dependent, and that individual microRNAs may either protect or exacerbate cardiovascular damage depending on the underlying stress condition and cellular environment [59,60].

From a translational perspective, microRNAs involved in oxidative stress and mitochondrial dysfunction are increasingly being considered as both biomarkers of subcellular injury and potential therapeutic targets. However, their clinical exploitation still faces important challenges, including disease- and cell-type specificity, incomplete understanding of mitomiR trafficking, and the difficulty of achieving efficient and selective mitochondrial delivery. Nevertheless, the rapid expansion of mitochondria-focused RNA research suggests that these molecules may open new opportunities for mechanism-based cardiovascular diagnostics and therapy [61,62].

### 4.3. Apoptosis and Cell Survival

Apoptosis and cell survival are fundamental determinants of cardiovascular tissue integrity, particularly under conditions of ischemia, inflammation, hemodynamic overload, and metabolic stress. In cardiovascular disease, an imbalance between pro-survival and pro-death signaling contributes to the progressive loss of cardiomyocytes, endothelial injury, plaque instability, and adverse remodeling. Within this context, microRNAs have emerged as critical modulators of cell fate because they fine-tune signaling pathways related to Bcl-2 family proteins, caspase activation, PI3K/AKT signaling, p53-dependent stress responses, and mitochondrial integrity [63,64].

Among the best-characterized anti-apoptotic microRNAs, miR-21 and miR-24 have received particular attention. Experimental studies have shown that miR-21 attenuates oxidative stress-induced cardiomyocyte apoptosis by targeting PDCD4 and related survival pathways, whereas miR-24 suppresses cardiomyocyte death by repressing pro-apoptotic mediators such as Bim and modulating intrinsic apoptotic signaling. These findings support the view that specific microRNAs actively preserve cardiovascular cell survival rather than merely reflecting injury-associated transcriptional changes [65,66].

By contrast, other microRNAs are more consistently associated with enhanced susceptibility to apoptosis. miR-34a, for example, has been implicated in ischemic and aging-related cardiac injury through the repression of prosurvival targets such as aldehyde dehydrogenase 2 (ALDH2), Notch1, and Bcl-2-related pathways, thereby favoring cardiomyocyte loss and maladaptive remodeling. In parallel, cardioprotective programs involving miR-133a have been linked to reduced apoptosis and improved cardiac function in experimental myocardial injury, underscoring the context-dependent and bidirectional nature of microRNA-mediated control over cell death and survival [67,68].

From a translational perspective, apoptosis-related microRNA signatures are increasingly being explored as both mechanistic biomarkers and therapeutic entry points in cardiovascular disease. Their appeal lies in the possibility of simultaneously capturing ongoing cellular injury and modulating upstream molecular pathways through mimics, inhibitors, or vector-based delivery strategies. Nevertheless, clinical application still faces significant obstacles, including cell-type specificity, temporal variability during disease progression, and the risk of off-target effects when targeting broadly interconnected survival pathways [69].

### 4.4. Fibrosis

Fibrosis is a central component of pathological cardiovascular remodeling and is characterized by excessive extracellular matrix deposition, persistent fibroblast activation, and progressive disruption of tissue architecture. In the heart and vasculature, this process compromises mechanical compliance, impairs electrical conduction, and favors the transition toward chronic dysfunction. At the molecular level, fibrotic remodeling is sustained by a complex network involving TGF-β/Smad signaling, inflammatory mediators, fibroblast–myofibroblast transition, and extracellular matrix gene reprogramming, all of which are increasingly recognized as being under microRNA control [70,71].

Among the most extensively studied antifibrotic regulators, the miR-29 family has emerged as a canonical suppressor of extracellular matrix production. Foundational work demonstrated that miR-29 directly targets transcripts encoding collagens, fibrillins, and elastin, and that its downregulation after myocardial infarction derepresses a broad profibrotic gene program. By contrast, miR-21 is widely regarded as a prototypical profibrotic microRNA because it promotes fibroblast activation and amplifies fibrogenic signaling through pathways involving TGF-β, ERK-MAPK, PI3K/Akt, and related mediators. Together, these molecules exemplify how distinct microRNA programs can either restrain or potentiate fibrotic remodeling [31,32].

Additional microRNAs further illustrate the complexity of fibrosis-related regulation. For example, miR-101 has been shown to attenuate post-infarction cardiac fibrosis and improve cardiac performance by inhibiting profibrotic signaling, including pathways linked to c-Fos and TGF-β-dependent responses. More broadly, recent analyses support the concept that fibrosis in cardiovascular disease cannot be interpreted as an isolated extracellular matrix phenomenon, but rather as part of a broader fibroinflammatory network in which microRNAs coordinate crosstalk among fibroblasts, immune cells, and injured parenchymal cells. This systems-level view helps explain why individual microRNAs may exert context-dependent effects depending on disease stage, cellular source, and the surrounding inflammatory milieu [72,73].

From a translational perspective, fibrosis-associated microRNAs are attractive both as candidate biomarkers of ongoing remodeling and as potential therapeutic targets. Their appeal lies in the possibility of capturing dynamic changes in collagen deposition, fibroblast activation, and maladaptive remodeling before irreversible structural deterioration becomes clinically evident. At the same time, therapeutic modulation of profibrotic or antifibrotic microRNAs remains challenging because of tissue specificity, delivery barriers, and the risk of off-target effects within highly interconnected signaling networks. Even so, growing progress in RNA-based cardiovascular therapeutics supports the view that fibrosis-related microRNAs may become increasingly relevant for future mechanism-based diagnostics and intervention strategies [74,75].

### 4.5. Angiogenesis and Vascular Dysfunction

Angiogenesis and vascular function are essential for the maintenance of tissue perfusion, oxygen delivery, and endothelial integrity in the cardiovascular system. Under physiological conditions, the vascular endothelium coordinates these processes through tightly regulated signaling pathways that govern proliferation, migration, barrier function, nitric oxide production, and communication with mural and immune cells. However, in cardiovascular disease, this homeostatic balance becomes disrupted, leading to endothelial dysfunction, impaired reparative angiogenesis, abnormal neovessel formation, and progressive vascular instability [13].

MicroRNAs have emerged as central post-transcriptional regulators of angiogenic and endothelial programs because they fine-tune VEGF signaling, inflammatory activation, redox balance, and endothelial responsiveness to biomechanical and metabolic stress. Their relevance is particularly evident in conditions such as atherosclerosis, ischemic injury, diabetic vasculopathy, and microvascular dysfunction, where changes in microRNA expression may alter both the structural and functional properties of the vascular compartment [7,8].

Among the best-characterized pro-angiogenic and vasculoprotective microRNAs, miR-126 remains a canonical example because it supports vascular integrity, enhances angiogenic signaling, and represses endothelial inflammatory activation. In parallel, miR-92a has been widely associated with impaired endothelial repair and defective angiogenesis, and its inhibition has been shown to improve vascular recovery in ischemic settings. These findings illustrate how specific microRNAs can either sustain endothelial homeostasis or promote vascular dysfunction depending on the direction of their dysregulation [9,35].

From a pathological perspective, angiogenesis-related microRNAs are also relevant because they connect endothelial dysfunction with broader remodeling processes, including inflammation, plaque progression, microvascular rarefaction, and impaired tissue regeneration. This broader view is particularly important in cardiovascular disease, where aberrant vascular remodeling is not limited to changes in vessel density but also involves qualitative defects in vascular integrity, permeability, and cellular communication. Consequently, microRNA signatures linked to angiogenesis and vascular dysfunction are increasingly being explored as both mechanistic biomarkers and candidate therapeutic targets in vascular disease [54,76].

### 4.6. Cardiac and Vascular Remodeling

Cardiac and vascular remodeling represent dynamic but ultimately maladaptive responses to persistent stress, including pressure overload, ischemia, inflammation, metabolic disturbance, and neurohumoral activation. Although remodeling initially serves to maintain tissue integrity and organ performance, sustained structural and molecular alterations progressively impair ventricular compliance, contractile function, vascular elasticity, and tissue perfusion. In cardiovascular disease, these changes are increasingly understood as the integrated output of deregulated signaling networks rather than as isolated consequences of cell injury, and microRNAs have emerged as important coordinators of this remodeling continuum [27,75].

In the myocardium, microRNAs contribute to remodeling by regulating cardiomyocyte hypertrophy, fibroblast activation, extracellular matrix turnover, and electrical adaptation. Among the best-characterized examples, miR-208a has been described as a major cardiac muscle-enriched regulator of hypertrophic growth and remodeling, whereas therapeutic inhibition of miR-208a has been shown to attenuate pathological remodeling and improve cardiac performance in experimental heart failure. These findings illustrate how individual microRNAs can operate as nodal regulators of remodeling programs rather than as isolated markers of disease progression [25,77].

In the vasculature, remodeling is driven by coordinated changes in endothelial behavior, vascular smooth muscle cell plasticity, extracellular matrix composition, and biomechanical adaptation. Recent evidence indicates that microRNAs participate in these responses by linking cyclic stretch, oxidative stress, inflammatory activation, and phenotypic switching to structural reorganization of the vessel wall. This is particularly relevant in atherosclerosis and other chronic vascular disorders, where microRNA-dependent pathways shape lesion progression, wall thickening, stiffness, and maladaptive repair [78].

**Figure 2:**
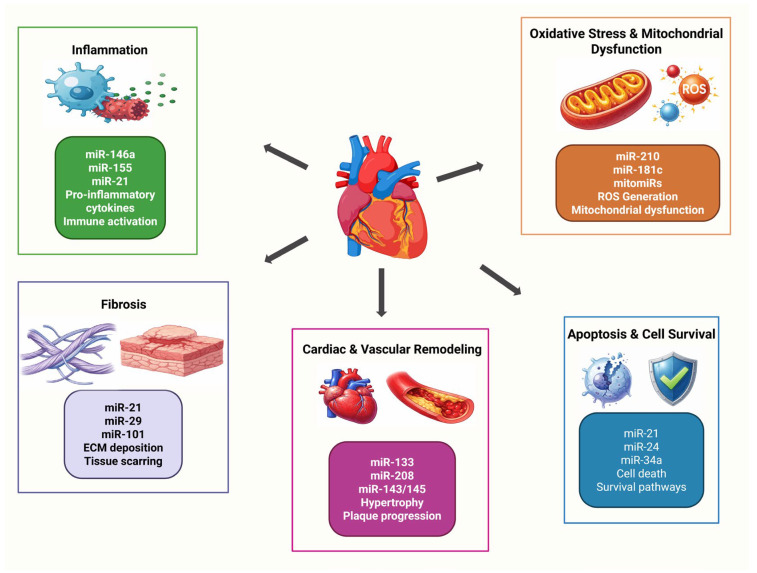
Representative microRNA-regulated molecular networks involved in cardiovascular pathology. The scheme summarizes major pathogenic processes regulated by microRNAs across cardiovascular disease, including inflammation, oxidative stress and mitochondrial dysfunction, apoptosis and cell survival, fibrosis, and cardiac and vascular remodeling. Representative microRNAs are shown as modulators of inflammatory signaling, redox imbalance, mitochondrial dysfunction, cell death and survival pathways, extracellular matrix deposition, tissue scarring, hypertrophy, and plaque progression. Together, these interconnected processes illustrate how microRNAs contribute to pathological cardiovascular remodeling. Created in BioRender. Vélez Slimani, H. (2026) https://BioRender.com/kigecea (accessed on 14 January 2026).

From a translational perspective, remodeling-associated microRNA signatures are increasingly viewed as informative indicators of disease progression and potential therapeutic entry points. Their value lies in the possibility of capturing the molecular transition from adaptive compensation to irreversible structural damage, while also offering opportunities for targeted intervention through mimics, inhibitors, or anti-miR strategies. However, successful clinical implementation still depends on improving tissue specificity, delivery efficiency, temporal profiling, and the interpretation of context-dependent microRNA networks across diverse remodeling phenotypes [54,79]. The major microRNA-regulated molecular networks involved in cardiovascular pathology, including inflammation, oxidative stress and mitochondrial dysfunction, apoptosis and cell survival, fibrosis, and cardiac and vascular remodeling, are summarized in Figure 2.

## 5. MicroRNAs in Specific Cardiovascular Diseases

While the previous section focused on shared molecular networks, the pathological relevance of microRNAs becomes even more evident when these regulatory programs are examined within specific cardiovascular diseases. In clinical settings, microRNA dysregulation does not occur in isolation but is embedded in distinct disease contexts characterized by different combinations of lipid imbalance, endothelial dysfunction, inflammation, fibrosis, hemodynamic stress, ischemic injury, and electrical remodeling. For this reason, disease-oriented analysis is essential to understand how microRNAs contribute to pathogenesis, reflect disease stage, and potentially support more precise diagnostic and therapeutic strategies across the cardiovascular spectrum [4,54].

### 5.1. Atherosclerosis

Atherosclerosis is a chronic inflammatory disease of the arterial wall driven by endothelial dysfunction, lipid accumulation, maladaptive immune activation, and progressive plaque remodeling. Rather than representing a purely passive storage disorder, it involves highly coordinated interactions among endothelial cells, macrophages, vascular smooth muscle cells, and extracellular matrix components, which together determine plaque growth, composition, and stability. Within this complex environment, microRNAs have emerged as key regulators of atherogenesis because they modulate the cellular crosstalk that links inflammation, cholesterol handling, phenotypic switching, and vascular injury [80,81].

At the endothelial level, microRNAs are critically involved in the early stages of atherogenesis by controlling inflammatory activation, leukocyte adhesion, and barrier integrity. Among them, miR-126 remains one of the most representative vasculoprotective examples, as it restrains endothelial activation through repression of VCAM-1 and supports vascular integrity. Loss or dysregulation of endothelial protective microRNAs may therefore facilitate monocyte recruitment and create a permissive environment for plaque initiation [11,13].

Macrophage-associated microRNAs further shape plaque development by regulating lipid metabolism, foam-cell formation, inflammatory polarization, and lesion progression. A well-established example is miR-33, which limits cholesterol efflux by targeting pathways upstream and downstream of ATP-binding cassette transporter A1 (ABCA1), thereby promoting intracellular lipid retention and impairing reverse cholesterol transport. In contrast, anti-miR-33 strategies have been shown to enhance cholesterol efflux and reduce atherosclerotic burden in experimental models, highlighting the functional importance of microRNA-mediated control of macrophage lipid handling [82,83].

Other microRNAs, such as miR-155 and miR-21, illustrate the context-dependent nature of microRNA signaling in atherosclerosis. miR-155 has been strongly linked to inflammatory macrophage activation and advanced lesion progression, although its effects may vary according to disease stage and plaque environment. miR-21, in turn, has been associated with both plaque growth and plaque stability, reflecting its pleiotropic influence on macrophages, smooth muscle cells, extracellular matrix turnover, and endothelial behavior. These examples underscore that microRNAs in atherosclerosis rarely function as isolated on/off switches; instead, they act within dynamic and cell-specific regulatory networks that influence both plaque progression and vulnerability [84,85].

From a translational perspective, atherosclerosis-associated microRNAs are increasingly being explored as candidate biomarkers and therapeutic targets. Their appeal lies in the possibility of capturing biologically meaningful changes related to endothelial injury, inflammatory burden, lipid dysregulation, and plaque instability, while also offering opportunities for intervention through mimics, inhibitors, and targeted delivery systems. However, major challenges remain, including inter-study variability, cell- and stage-specific effects, and the difficulty of distinguishing causal regulatory microRNAs from those that merely reflect ongoing vascular damage [54,86].

### 5.2. Hypertension

Hypertension is a multifactorial disorder in which sustained elevation of blood pressure arises from the interaction of vascular dysfunction, renal dysregulation, neurohumoral activation, inflammation, and structural remodeling. Rather than being explained by a single pathogenic axis, hypertension reflects the cumulative disruption of interconnected regulatory systems that control vascular tone, sodium balance, endothelial function, and arterial stiffness. In this context, microRNAs have emerged as important modulators of blood pressure regulation because they influence molecular pathways operating in the vasculature, kidneys, immune system, and renin–angiotensin–aldosterone system [87,88].

At the vascular level, microRNAs contribute to hypertension by shaping endothelial dysfunction, smooth muscle cell behavior, and vessel wall remodeling. Endothelial- and vascular-associated microRNAs regulate nitric oxide signaling, oxidative stress, inflammatory activation, and phenotypic switching, thereby influencing arterial stiffness and maladaptive vascular responses. This is particularly relevant because hypertension is both a cause and a consequence of endothelial dysfunction, creating a self-reinforcing cycle in which altered microRNA expression may amplify vascular injury and impair adaptive homeostatic mechanisms [89,90].

Beyond the vasculature, several microRNAs participate in neurohumoral and renal mechanisms of hypertension. A representative example is miR-181a, which has been linked to renin regulation in the kidney; reduced renal miR-181a expression has been associated with increased renin abundance and elevated blood pressure, supporting the idea that microRNA dysregulation may contribute directly to activation of the renin–angiotensin system. In parallel, other microRNAs, including miR-21 and miR-155, have been implicated in hypertensive remodeling, inflammatory signaling, vascular dysfunction, and target-organ damage, although their biological effects may vary according to tissue, disease stage, and experimental context [91,92,93].

From a translational perspective, microRNAs associated with hypertension are increasingly being explored as circulating biomarkers and as potential therapeutic targets. Recent reviews emphasize that circulatory miRNAs may provide information on vascular remodeling, endothelial dysfunction, and disease subtype, although current evidence is still limited by methodological heterogeneity, small cohorts, and incomplete reproducibility across studies. Even so, the growing integration of mechanistic, biomarker, and therapeutic research suggests that microRNA-based approaches may eventually contribute to more precise risk stratification and individualized management of hypertensive disease [94,95].

### 5.3. Acute Myocardial Infarction

Acute myocardial infarction (AMI) is characterized by abrupt coronary artery occlusion leading to myocardial ischemia, irreversible cardiomyocyte death, and the subsequent activation of inflammatory and reparative responses. Although advances in reperfusion therapy and diagnostic algorithms have markedly improved short-term outcomes, AMI remains a major cause of mortality and long-term disability because the initial ischemic insult is frequently followed by adverse ventricular remodeling and progressive functional decline. In this setting, microRNAs have emerged as relevant disease-associated regulators because they participate in the molecular transition from acute ischemic injury to tissue repair and remodeling by modulating inflammation, oxidative stress, mitochondrial dysfunction, and cell death pathways [64,96].

One of the most clinically attractive aspects of microRNA biology in AMI is the rapid appearance of cardiac-enriched species in the circulation after myocardial injury. Circulating miR-1, miR-133a, miR-208a/b, and miR-499 have been repeatedly associated with AMI and are considered promising complementary biomarkers because they reflect myocardial damage with high tissue relevance and may rise early after symptom onset. This has generated sustained interest in their use as adjuncts to high-sensitivity cardiac troponins, particularly in the earliest diagnostic window and in risk stratification settings where conventional biomarkers may be less informative [54,97].

Beyond their biomarker value, several microRNAs appear to influence the biological response to infarction in a context-dependent manner. miR-24, for example, has been described as a protective regulator capable of attenuating oxidative stress, inflammation, apoptosis, and fibrotic remodeling in ischemic cardiac injury, whereas miR-21 is increasingly viewed as a double-edged molecule that may provide cardioprotection during acute ischemic stress but also contribute to later fibrosis and maladaptive remodeling if persistently dysregulated. These observations illustrate that microRNAs in AMI should not be interpreted simply as passive markers of tissue damage, but rather as active components of the injury–repair continuum [98,99].

An additional clinically important dimension of AMI is the emergence of post-ischemic ventricular arrhythmias, which remain a major cause of early mortality and may also contribute to later sudden cardiac death. These rhythm disturbances arise from a complex interaction among ischemia-driven electrical instability, gap-junction remodeling, inflammatory activation, and autonomic remodeling [100,101]. Within this setting, miR-1 and other arrhythmia-associated microRNAs have been linked to altered excitability, conduction abnormalities, and maladaptive post-infarction remodeling [39]. In parallel, inflammatory microRNAs such as miR-155 appear to connect innate immune activation with ventricular arrhythmogenic remodeling, as their inhibition has been shown to attenuate connexin 43 degradation and sympathetic neural remodeling after myocardial infarction by reducing pro-inflammatory macrophage activation [102]. These observations reinforce the concept that post-infarction arrhythmogenesis should be interpreted not only as an electrophysiological consequence of ischemic injury, but also as an inflammatory and microRNA-regulated process.

From a translational standpoint, microRNAs associated with AMI are increasingly being explored as tools for precision cardiology, particularly for early diagnosis, post-infarction risk stratification, and the identification of patients at risk of adverse remodeling. However, substantial barriers remain before routine clinical implementation can be achieved, including pre-analytical variability, lack of standardized normalization strategies, inconsistent findings across cohorts, and the difficulty of distinguishing acute injury-associated signatures from those related to subsequent remodeling stages. Even so, the continued integration of mechanistic and clinical research suggests that microRNA-based approaches may become increasingly relevant in the future management of AMI [103,104].

### 5.4. Heart Failure

Heart failure (HF) represents the final common clinical phenotype of multiple cardiovascular disorders and is characterized by the progressive inability of the heart to maintain adequate systemic perfusion or to do so only at the expense of elevated filling pressures. Although HF is clinically heterogeneous, its progression is generally sustained by interconnected processes that include cardiomyocyte stress, inflammation, fibrosis, metabolic reprogramming, maladaptive hypertrophy, and ventricular remodeling. Within this framework, microRNAs have emerged as relevant regulators of HF pathophysiology because they modulate the molecular networks that connect structural remodeling with functional deterioration [69,105].

From a mechanistic perspective, microRNAs contribute to heart failure by influencing hypertrophic signaling, extracellular matrix remodeling, mitochondrial stress responses, calcium handling, and inflammatory activation. Rather than functioning as isolated markers, they appear to participate in broader regulatory circuits that shape disease progression and treatment response. This systems-level role is particularly relevant in HF, where small changes in coordinated gene expression programs may determine whether the myocardium remains compensatory or progresses toward decompensation [69,106].

Heart failure with preserved ejection fraction (HFpEF) deserves specific consideration because its pathophysiology is not fully captured by frameworks centered on reduced ejection fraction. In HFpEF, microRNA dysregulation appears to be closely linked to chronic low-grade inflammation, endothelial and microvascular dysfunction, myocardial stiffness, and fibroinflammatory remodeling, all of which contribute to impaired relaxation and diastolic dysfunction. Rather than reflecting a purely cardiomyocyte-centered disorder, HFpEF is increasingly understood as a multisystem syndrome in which comorbidity-driven inflammation and vascular dysfunction interact with extracellular matrix remodeling and altered myocardial signaling. Within this context, microRNAs are of particular interest because they may help connect inflammatory and fibrotic mechanisms with phenotype-specific biomarker development and emerging precision-medicine strategies in HFpEF [73,107].

One of the most intensely explored clinical applications of microRNAs in HF is their use as circulating biomarkers. Among them, miR-423-5p has attracted sustained attention because elevated circulating levels have been associated with HF diagnosis and disease severity, and more recent evidence suggests that combining miR-423-5p with conventional measures such as BNP and left ventricular ejection fraction may improve diagnostic performance. In addition, recent work on circulating miRNA panels indicates that composite signatures may provide prognostic and risk-stratification value beyond single-marker approaches, particularly when distinguishing between HF phenotypes and progression trajectories [107,108].

From a therapeutic standpoint, heart failure is also one of the most advanced cardiovascular settings for the clinical translation of microRNA-targeted interventions. Inhibition of miR-132 has emerged as a promising strategy because pathologically increased miR-132 is linked to adverse cardiac remodeling, and the antisense oligonucleotide CDR132L has already progressed from preclinical studies to early-phase clinical evaluation. In parallel, emerging preclinical evidence suggests that HFpEF may also be therapeutically approachable through microRNA modulation, as inhibition of miR-92a has been shown to normalize vascular gene expression and prevent diastolic dysfunction in experimental HFpEF, supporting the idea that phenotype-specific microRNA targeting may become relevant beyond HFrEF-oriented strategies [109].

Despite this promise, several obstacles still limit the routine implementation of microRNA-based strategies in heart failure, including variability across cohorts, incomplete standardization of sampling and normalization methods, disease-stage specificity, and the complexity of interpreting tissue-derived versus circulating signatures. Nevertheless, the rapid convergence of mechanistic, biomarker, and therapeutic studies strongly supports the view that microRNAs may become increasingly relevant for precision medicine approaches in HF, particularly when integrated with conventional clinical and imaging parameters [110].

### 5.5. Arrhythmias

Cardiac arrhythmias arise from complex interactions among electrical remodeling, structural remodeling, altered calcium handling, conduction abnormalities, and autonomic influences. In this context, microRNAs have gained increasing attention because they regulate multiple determinants of arrhythmogenesis, including ion channel expression, gap junction integrity, fibrosis, and inflammatory signaling. Rather than acting as isolated factors, these molecules appear to organize broader regulatory networks that can either preserve electrophysiological stability or promote proarrhythmic substrates under pathological conditions [39,111].

Among rhythm disorders, atrial fibrillation (AF) is the most intensively studied in relation to microRNAs, although these regulators are also increasingly recognized in post-ischemic ventricular arrhythmogenesis. Current evidence indicates that several microRNAs participate in both electrical and structural atrial remodeling, thereby influencing AF initiation, maintenance, and progression. MiR-1 has been linked to conduction and ion-channel remodeling, while miR-328 has been associated with altered calcium-channel regulation and increased AF susceptibility. These observations support the view that microRNA dysregulation contributes directly to arrhythmogenic remodeling rather than merely reflecting its downstream consequences [111,112,113].

Structural remodeling is equally important in arrhythmia biology, especially in AF, where fibrosis promotes conduction heterogeneity and re-entry. In this setting, miR-21 has emerged as a representative profibrotic regulator because its upregulation is associated with reduced Spry1 expression, increased collagen-related signaling, and enhanced atrial fibrosis. This reinforces the concept that arrhythmia-associated microRNAs not only affect electrophysiology, but also shape the underlying substrate by modulating fibroblast activation and extracellular matrix remodeling [114,115].

These regulatory mechanisms are also highly relevant to ventricular arrhythmias, particularly after myocardial infarction. Post-infarction ventricular tachycardia and ventricular fibrillation are shaped not only by scar-related conduction slowing and ion-channel dysregulation, but also by inflammatory cytokine signaling, macrophage activation, connexin remodeling, and sympathetic neural reorganization [100,101]. In this context, miR-1 remains a representative arrhythmogenic microRNA linked to conduction abnormalities, whereas inflammatory microRNAs such as miR-155 provide a mechanistic bridge between innate immune activation and ventricular electrical instability [39,102]. Expanding the arrhythmia framework beyond atrial fibrillation, therefore, helps integrate ischemic injury, inflammation, and microRNA dysregulation into a more comprehensive view of cardiovascular arrhythmogenesis.

Arrhythmogenesis is also increasingly understood as a process shaped by inflammatory signaling and innate immune activation. In both atrial and ventricular myocardium, cytokine release and immune-cell recruitment can promote ion-channel dysregulation, connexin remodeling, fibroblast activation, and conduction heterogeneity, thereby facilitating arrhythmia initiation and maintenance [100,101]. In atrial fibrillation, inflammation-associated microRNA programs have been linked to atrial enlargement, hsCRP, and fibrotic remodeling, with increased miR-146b-5p and miR-155 among the reported changes [116]. This inflammatory–electrophysiological interface also appears relevant after myocardial infarction, where miR-155-dependent macrophage activation has been associated with connexin 43 degradation, sympathetic neural remodeling, and greater ventricular arrhythmia susceptibility [102].

From a translational standpoint, microRNAs are increasingly being investigated as biomarkers for arrhythmia diagnosis, recurrence prediction, and patient stratification. Recent studies suggest that circulating and extracellular RNA signatures may provide information on atrial remodeling and AF burden, while post-ablation and postoperative settings have also generated interest in miRNA-based predictive models. Nevertheless, major obstacles remain before routine implementation can be achieved, including low disease specificity, inconsistent findings across cohorts, limited standardization, and uncertainty regarding which miRNAs are causal drivers rather than secondary markers of remodeling [115,117,118,119].

## 6. MicroRNAs as Clinical Biomarkers

Interest in microRNAs as cardiovascular biomarkers stems from their relevance as regulators of pathophysiological pathways and as measurable circulating molecules in accessible biological fluids. In contrast to conventional biomarkers that often reflect a restricted aspect of tissue injury, circulating microRNAs may capture broader processes such as endothelial dysfunction, inflammation, oxidative stress, metabolic disturbance, and maladaptive remodeling. This has positioned them as attractive candidates for early detection, disease stratification, prognostic assessment, and therapeutic monitoring in cardiovascular medicine [120,121].

At the same time, the translation of circulating microRNAs into routine clinical practice remains challenging. Recent reviews emphasize that methodological variability in sample collection, processing, storage, normalization, and quantification continues to limit reproducibility across studies, even though these molecules show considerable promise as blood-based biomarkers. As a result, the biomarker field has increasingly shifted from simple discovery efforts toward standardization, multicohort validation, and the identification of disease-specific signatures with stronger clinical robustness [122].

### 6.1. Circulating MicroRNAs in Plasma and Serum

Among the different biospecimens explored in cardiovascular biomarker research, plasma and serum remain the most widely used sources for circulating microRNA analysis because they are minimally invasive, clinically accessible, and suitable for repeated sampling. Their relevance is further supported by the relative stability of circulating microRNAs under a range of handling and storage conditions, which has strengthened their appeal as candidates for translational biomarker development in cardiovascular disease [121].

In ischemic heart disease and acute coronary syndromes, several circulating microRNAs have been repeatedly associated with disease presence, biological activity, and clinical severity. Recent evidence highlights recurrent dysregulation of miR-126, miR-21, miR-145, miR-92a, and miR-155 across ischemic heart disease subtypes, while cardiac- and muscle-enriched species such as miR-1, miR-133a, miR-133b, miR-208a, and miR-499 have been particularly linked to myocardial injury and proposed as promising diagnostic and prognostic biomarkers in acute coronary syndromes [123,124].

Despite this promise, plasma- and serum-based microRNA profiling still faces important analytical and interpretive limitations. Variability in blood collection tubes, hemolysis, centrifugation protocols, storage conditions, RNA extraction workflows, and normalization strategies can substantially affect measured microRNA levels and reduce inter-study comparability. Consequently, the clinical value of circulating microRNAs will depend not only on identifying disease-associated signatures, but also on establishing standardized pre-analytical and analytical pipelines capable of supporting reproducible large-scale validation [122,125,126].

An additional source of heterogeneity arises from the analytical platforms used for circulating microRNA measurement. Across cardiovascular studies, quantification has relied mainly on RT-qPCR, microarrays, next-generation sequencing, and, more recently, digital PCR-based approaches, each with distinct advantages and limitations in sensitivity, specificity, normalization, and absolute versus relative quantification. These technical differences complicate direct comparison across studies, especially when combined with variable endogenous or exogenous controls, inconsistent reporting of assay performance, and limited harmonization of cutoff definitions. Moreover, many proposed biomarker signatures derive from relatively small discovery cohorts and are not consistently replicated in independent or ethnically distinct populations, which further limits their generalizability and translational robustness. For this reason, future progress will depend not only on identifying disease-associated miRNAs, but also on improving methodological reporting, cohort design, and multicenter validation strategies [121,125,126].

### 6.2. Extracellular Vesicles and Exosomes

Extracellular vesicles (EVs), including exosomes, have emerged as highly relevant carriers of microRNAs in cardiovascular disease because they protect RNA cargo from degradation and mediate intercellular communication between endothelial cells, cardiomyocytes, fibroblasts, immune cells, and vascular smooth muscle cells. This feature makes EV-associated microRNAs particularly attractive from a biomarker perspective, as they may better reflect active cellular signaling and disease-specific biological processes than unfractionated circulating RNA alone [127,128].

From a clinical standpoint, EV-derived microRNAs are being actively investigated as minimally invasive biomarkers for diagnosis, prognosis, and risk stratification across cardiovascular settings. Recent evidence suggests that EV cargo may capture disease-relevant signatures linked to ischemic injury, heart failure progression, and adverse cardiovascular outcomes. For example, plasma extracellular vesicle cargo microRNAs have been associated with subsequent heart failure and cardiovascular death after acute coronary syndrome, reinforcing the idea that vesicle-associated miRNA profiles may carry prognostic information beyond conventional circulating markers [129,130].

Beyond biomarker discovery, EV- and exosome-associated microRNAs are also attracting major interest as therapeutic tools. Preclinical studies indicate that exosomal miRNA cargo can modulate inflammation, apoptosis, fibrosis, angiogenesis, and tissue repair, particularly in ischemic and remodeling-associated cardiovascular conditions. This has made EVs appealing as natural delivery systems for RNA-based therapy, especially when derived from stem or progenitor cell sources, although most evidence remains preclinical and substantial translational work is still required [131,132].

Despite these advantages, several limitations continue to hamper the clinical implementation of EV-derived microRNAs. Major challenges include the lack of standardized isolation and characterization protocols, variability in EV yield and purity, difficulties in assigning vesicles to their tissue of origin, and inconsistencies across analytical platforms. Consequently, the future clinical value of EV-associated microRNAs will depend not only on identifying biologically meaningful signatures, but also on establishing robust methodological frameworks that enable reproducible and clinically interpretable results [133].

### 6.3. Diagnostic, Prognostic, and Monitoring Value

The diagnostic, prognostic, and monitoring value of cardiovascular microRNAs lies in the fact that they do not merely indicate the presence of tissue injury, but may also reflect ongoing biological activity, remodeling dynamics, and response to therapy. This makes them particularly attractive in cardiovascular medicine, where disease evolution is often continuous rather than static. Recent studies further suggest that advances in RNA-focused biosensing may enhance the clinical usability of these molecules by enabling faster quantification, improved analytical sensitivity, and more practical real-time monitoring strategies [134,135].

The prognostic and stratification potential of microRNAs is increasingly supported by studies moving beyond single-candidate markers toward broader signatures and disease-oriented profiling. A large multicentre European study reported that circulating miRNA profiles may help classify major cardiovascular phenotypes, including acute coronary syndrome, chronic coronary artery disease, dilated cardiomyopathy, and ischemic cardiomyopathy. Likewise, recent analyses in heart failure support the diagnostic and prognostic relevance of circulating miRNAs and suggest that integrated miRNA-based approaches may improve patient stratification across heterogeneous clinical settings [136,137].

Their monitoring value is also becoming more evident in serial and time-resolved studies. Experimental work on cardiac ischemia and remodeling showed that dynamic miRNA signatures can track phases of myocardial injury and resolution, supporting their use as noninvasive indicators of disease evolution rather than one-time diagnostic markers. Likewise, in acute heart failure, serial in-hospital measurements demonstrated that persistently low miR-140-3p levels were associated with higher long-term mortality and more heart-failure events, illustrating how repeated miRNA assessment may help capture prognosis and treatment-related trajectory over time [138,139].

Despite this promise, clinical implementation still requires stronger standardization and more rigorous integration with conventional cardiovascular assessment. Broader biomarker reviews emphasize that the future value of microRNAs will likely depend on multimarker strategies combined with clinical data, imaging, and computational tools rather than on isolated readouts alone. Consistently, recent work in acute myocardial infarction suggests that integrating miRNA profiling with standard clinical evaluation may improve early diagnosis and personalize risk stratification, although further validation in larger and more diverse cohorts remains essential [140,141]. Representative microRNAs associated with major cardiovascular diseases, together with their reported expression patterns, principal pathological networks, and potential clinical relevance, are summarized in Table 2.

## 7. MicroRNAs as Therapeutic Tools

The therapeutic interest in microRNAs arises from their position as upstream regulators of complex gene networks rather than single downstream effectors. This gives them a particular advantage in cardiovascular disease, where pathological phenotypes such as inflammation, fibrosis, hypertrophy, endothelial dysfunction, and adverse remodeling are sustained by interconnected molecular programs. In this setting, therapeutic modulation of microRNAs has emerged as a promising strategy to either suppress pathologically overexpressed miRNAs or restore the activity of protective miRNAs that become depleted during disease progression [151,152].

At the same time, the development of microRNA-based therapeutics remains more challenging than conventional small-molecule pharmacology because of issues related to in vivo stability, tissue specificity, immune activation, off-target effects, and intracellular delivery. As a result, the translational field has increasingly focused not only on identifying disease-relevant miRNA targets, but also on refining the chemistry, formulation, and delivery platforms required for safe and effective cardiovascular application [153,154].

### 7.1. Mimics and Inhibitors

MicroRNA therapeutics are generally based on two complementary approaches: miRNA mimics, which aim to restore the function of downregulated protective microRNAs, and miRNA inhibitors, which are designed to silence pathologically upregulated microRNAs. Conceptually, this framework is particularly attractive in cardiovascular disease because it allows direct modulation of disease-driving regulatory circuits rather than isolated protein targets. However, the biological pleiotropy of microRNAs also means that both strategies require careful attention to dose, tissue targeting, and unintended effects on parallel pathways [153,155].

Among these two modalities, inhibitory strategies are currently the most advanced in cardiovascular translation. Antisense-based approaches targeting pathogenic microRNAs have shown encouraging results in preclinical and early clinical development, with antimiR-132 (CDR132L) representing the most mature cardiovascular example to date. CDR132L was developed to inhibit pathologically elevated miR-132, a microRNA implicated in adverse cardiac remodeling, and early clinical studies in chronic ischemic heart failure reported favorable safety, target engagement, and signals of functional cardiac improvement, supporting its continued evaluation in post-myocardial infarction heart failure settings [156,157].

By contrast, miRNA replacement strategies using mimics remain highly promising but somewhat less advanced clinically, largely because of the additional challenges associated with efficient tissue delivery and sustained functional uptake. Even so, recent cardiovascular studies provide proof of concept that mimic-based therapy can be effective when paired with appropriate carriers. A 2025 study showed that miR-26b mimic-loaded lipid nanoparticles reduced atherogenesis and improved inflammatory features in experimental atherosclerosis, while broader regenerative reviews have highlighted the therapeutic potential of proregenerative microRNAs for cardiac repair when delivered in a controlled and nonpermanent manner [158,159].

Overall, mimics and inhibitors should not be viewed as competing platforms, but rather as complementary therapeutic tools whose usefulness depends on the underlying disease biology and the direction of microRNA dysregulation. Their future success in cardiovascular medicine will likely depend on combining mechanistic target selection with optimized delivery systems, temporal control of expression, and careful evaluation of safety across distinct cardiovascular phenotypes [74,154].

### 7.2. Antagomirs and Silencing Strategies

Antagomirs and related antisense-based silencing strategies are designed to inhibit pathogenic microRNAs through sequence-complementary binding, thereby preventing their interaction with target mRNAs and promoting functional derepression of disease-relevant genes. In cardiovascular disease, this approach is particularly attractive when a given microRNA is persistently overexpressed and contributes to maladaptive remodeling, endothelial dysfunction, inflammatory activation, or lipid imbalance. Advances in oligonucleotide chemistry, including locked nucleic acid (LNA), phosphorothioate backbone modifications, and other stability-enhancing designs, have substantially improved the pharmacological feasibility of microRNA silencing in vivo [153,160].

Preclinical studies have provided strong proof of concept that antagomir-based inhibition can beneficially modulate cardiovascular phenotypes. A representative recent example is anti-miR-92a, where LNA-based inhibition normalized vascular gene expression and attenuated diastolic dysfunction and left atrial enlargement in experimental HFpEF. Likewise, the miR-33 axis continues to illustrate the therapeutic logic of silencing overexpressed microRNAs, since inhibition of miR-33 has been associated with improved cholesterol efflux, enhanced reverse cholesterol transport, and anti-atherosclerotic effects in cardiovascular and metabolic disease models [109,161].

Among cardiovascular antagomirs, anti-miR-92a is especially relevant from a translational perspective because it has moved beyond small-animal proof-of-concept into human-oriented development. Reviews of the field note that MRG-110, an LNA-based anti-miR-92a compound, entered early-phase clinical testing and showed target derepression in human blood compartments, supporting the feasibility of microRNA silencing in a clinical context. These observations are important because they indicate that antagomir-based therapy is no longer only theoretical in cardiovascular medicine, but has already reached an early translational stage [154,162].

Despite this progress, several challenges still limit the broader clinical application of antagomirs. Efficient tissue-selective delivery remains a major bottleneck, particularly for the heart and vascular wall, and systemic exposure raises concerns regarding off-target effects, immune activation, and unintended modulation of miRNA networks in noncardiovascular tissues. In addition, the species-specific biology of some microRNAs complicates preclinical modeling and may affect how well experimental findings translate into humans. For these reasons, the future success of antagomir-based silencing strategies will depend not only on identifying robust disease-driving targets, but also on improving delivery technologies and minimizing unintended biological effects [163,164].

### 7.3. Delivery Systems

The development of microRNA-based therapies for cardiovascular diseases depends largely on delivery systems capable of protecting these molecules from degradation, prolonging their circulation time, and promoting their accumulation in target tissues. In contrast to naked microRNAs, delivery vehicles can improve stability, cellular uptake, and biodistribution, which is especially relevant in the heart and vasculature, where anatomical and physiological barriers limit therapeutic efficiency. In this context, delivery systems should be viewed not only as pharmaceutical platforms, but also as critical determinants of tissue specificity and the clinical translation of RNA therapeutics [165,166].

Among non-viral strategies, lipid, polymeric, and inorganic nanoparticles have attracted particular attention because of their ability to encapsulate mimics or inhibitors, facilitate cellular internalization, and modulate therapeutic cargo release [166]. In addition, platforms such as injectable hydrogels and hybrid nanoparticle-hydrogel systems have emerged as attractive alternatives to improve intramyocardial retention and reduce the rapid loss of therapeutic material after administration [167]. Preclinical studies have shown that these systems can direct therapeutic microRNAs toward injured tissues and improve remodeling and cardiac function, as described for miR-21 in post-infarction cardiac macrophages [168], miR-133 in infarct lesions [169], and miR-199a-5p in ischemic myocardium [170].

Extracellular vesicles and exosomes, in turn, represent particularly promising biomimetic systems, as they provide biocompatibility, cargo protection, and a natural capacity for intercellular communication [171]. Their bioengineering can enhance microRNA loading and optimize tissue targeting, although their application still faces important limitations related to vesicle heterogeneity, isolation standardization, and scalability. Finally, more sophisticated approaches based on active targeting and physically assisted release are also emerging, such as lipid nanoparticles combined with ultrasound-induced cavitation, with the aim of increasing treatment precision in cardiovascular settings [172]. Delivery systems are therefore critical for improving tissue targeting, therapeutic persistence, and translational feasibility in cardiovascular microRNA therapy.

### 7.4. Translational Limitations

Despite notable preclinical progress, the clinical translation of microRNA-based therapies in cardiovascular disease still faces major barriers. One of the main limitations arises from microRNA biology itself: because a single microRNA can modulate multiple transcripts and signaling pathways, its therapeutic manipulation may produce broad, difficult-to-predict, and potentially unwanted biological effects. Accordingly, specificity, selectivity, the definition of safe therapeutic windows, and the comprehensive assessment of adverse effects remain central challenges for the clinical development of this strategy [155,173].

Another critical limitation is efficient and reproducible delivery to cardiovascular tissue. Therapeutic oligonucleotides must overcome nuclease degradation, systemic clearance, heterogeneous cellular uptake, and intracellular barriers that restrict effective activity. Even when internalization occurs, endosomal entrapment remains a major bottleneck because it reduces the fraction of the molecule that actually reaches its site of action. In cardiovascular settings, this issue is especially relevant because platforms that perform well in other organs do not necessarily achieve the same efficacy in the heart and vasculature [174,175].

In addition, systemic safety remains a major concern. Clinical experience from the broader microRNA therapeutics field has shown that immune-mediated toxicity, unintended immune activation, and off-target effects can compromise clinical advancement even when biological activity is demonstrated. In this regard, the clinical development of MRX34, a liposomal miR-34a mimic evaluated in oncology, clearly illustrated how immune-mediated adverse events can limit the continuation of microRNA-based therapeutic programs, even when pharmacodynamic proof of concept exists [176].

**Figure 3:**
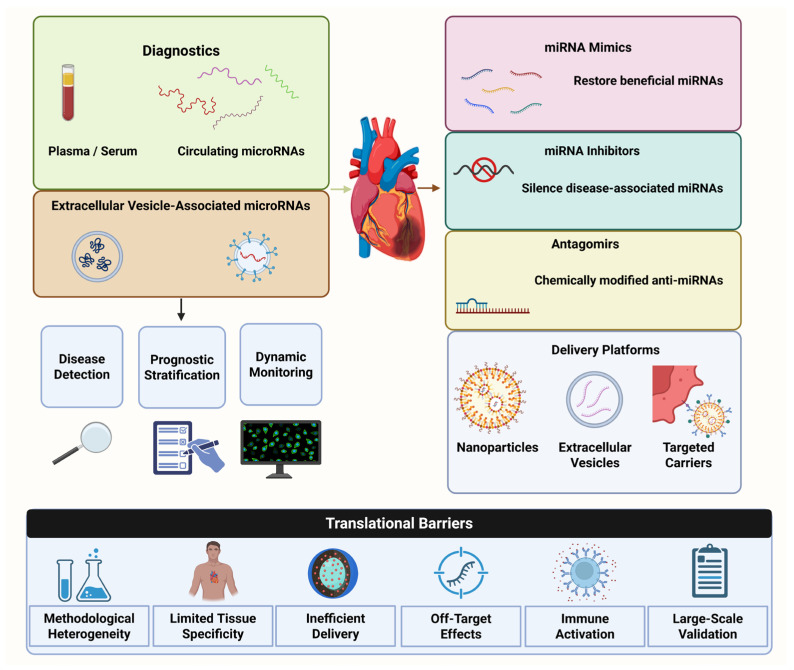
Diagnostic and therapeutic potential of cardiovascular microRNAs. The scheme summarizes the translational landscape of cardiovascular microRNAs, including diagnostic applications, therapeutic strategies, and major barriers to clinical implementation. On the diagnostic side, circulating and extracellular vesicle-associated microRNAs are presented as candidate tools for disease detection, prognostic stratification, and dynamic monitoring. On the therapeutic side, the main approaches under investigation include miRNA mimics, miRNA inhibitors, antagomirs, and selected delivery platforms. The figure also highlights major translational barriers, including methodological heterogeneity, limited tissue specificity, inefficient delivery, off-target effects, immune activation, and the need for large-scale validation. Created in BioRender. Vélez Slimani, H. (2026) https://BioRender.com/2mpgo51 (accessed on 14 January 2026).

Finally, translational limitations are not only biological but also regulatory and manufacturing-related. Recent FDA guidance for oligonucleotide therapeutics emphasizes the need to characterize immunogenicity, drug interactions, and the impact of hepatic and renal impairment during clinical development. In parallel, the FDA has indicated that products containing nanomaterials require specific characterization of quality attributes and chemistry, manufacturing, and controls (CMC) controls, while the EMA has established detailed considerations for the manufacturing process, characterization, specifications, and analytical control of synthetic oligonucleotides. Overall, this indicates that the clinical feasibility of microRNA therapies will depend not only on experimental efficacy, but also on scalable platforms, reproducible batches, and robust regulatory frameworks [177,178,179]. The diagnostic and therapeutic potential of microRNAs in cardiovascular diseases, including their roles as circulating biomarkers, extracellular vesicle-associated microRNAs, and targets for RNA-based interventions, is summarized in Figure 3.

## 8. Current Challenges and Future Perspectives

Future progress in cardiovascular microRNA research will depend on reducing methodological heterogeneity, improving biological resolution, and strengthening multicohort clinical validation. One of the main problems is methodological heterogeneity across studies, including differences in sample type, preanalytical processing, normalization strategies, analytical platforms, and reporting criteria. In this setting, reproducibility remains a critical challenge, particularly in RT-qPCR-based studies and extracellular vesicle analyses, where the MIQE 2.0 and MISEV2023 guidelines reinforce the need for more rigorous, comparable, and transparent workflows [180,181,182]. These limitations are compounded by the frequent use of small or single-center cohorts and by the still limited replication of candidate signatures across independent populations, factors that continue to weaken clinical transferability. Another important limitation is that the strength of evidence remains uneven across the field, with some conclusions supported by replicated human data and mechanistic validation, whereas others still rely primarily on exploratory or preclinical findings. Representative studies and their qualitative appraisal are summarized in Supplementary Table S1.

Another central challenge is biological specificity. Many microRNA profiles derived from peripheral blood or bulk tissue do not accurately capture the cellular complexity of the cardiovascular system, since they integrate signals from multiple cell types, functional states, and pathological contexts. For this reason, a particularly promising future direction will be to combine microRNA research with single-cell transcriptomics, spatial transcriptomics, and computational tools capable of inferring microRNA activity at cellular and spatial resolution. These approaches may help distinguish regulatory programs specific to cardiomyocytes, fibroblasts, endothelial cells, vascular smooth muscle cells, and immune cells, thereby refining both mechanistic interpretation and the selection of biomarkers and therapeutic targets [183,184,185].

From a clinical perspective, the future of the field will likely depend less on individual microRNAs and more on multimolecular signatures integrated with clinical variables, imaging, and other omics layers. In this regard, multi-omics and machine learning approaches are gaining importance because they can handle high-dimensional data, identify cardiovascular subphenotypes, and build more robust stratification models. However, for these strategies to become truly useful in clinical practice, they will need to be validated in prospective, multicenter, and well-phenotyped cohorts, while also moving toward harmonized thresholds and more standardized analytical frameworks [186,187].

Taken together, progress in the field will require a shift from descriptive associations toward integrated, reproducible, and biologically resolved models. The next step will be to define the cellular contexts in which microRNAs act, how they change over time, and how they may be incorporated more reliably into precision diagnostics, monitoring strategies, and targeted therapies.

## 9. Conclusions

MicroRNAs are increasingly recognized as important regulators of cardiovascular biology, linking cellular homeostasis with inflammation, stress responses, cell death, and pathological remodeling. Through their actions in endothelial cells, vascular smooth muscle cells, cardiomyocytes, and cardiac fibroblasts, these small non-coding molecules appear to modulate processes relevant to cardiovascular maintenance and disease progression, including atherosclerosis, hypertension, acute myocardial infarction, heart failure, and arrhythmias. Beyond their mechanistic relevance, microRNAs also represent promising candidates for biomarker and therapeutic development, although their clinical utility remains constrained by between-study heterogeneity, methodological standardization, tissue specificity, safety considerations, and the need for validation in well-characterized cohorts. Overall, current evidence suggests that microRNAs are best understood not as isolated regulators, but as components of complex biological networks linking cardiovascular physiology with disease and potential clinical application.

## Figures and Tables

**Table 1:** Relevant microRNAs by cardiovascular cell type and biological function.

Cardiovascular Cell Type	microRNA	Main Homeostatic Function	Representative Targets/Pathways	Consequence of Dysregulation	Experimental Context/Species	Reference
Endothelial cells	miR-126	Maintains vascular integrity, endothelial repair, and angiogenic competence	VEGF-related signaling; *SPRED1*/*PIK3R2*; VCAM-1-associated endothelial activation	Endothelial dysfunction, impaired angiogenesis, and enhanced vascular inflammation	Predominantly human and murine endothelial/vascular models	[34]
Endothelial cells	miR-92a	Regulates endothelial quiescence, repair capacity, and angiogenic responsiveness	Pro-angiogenic signaling networks in endothelial cells	Defective neovascularization and impaired functional vascular recovery after ischemia	Mouse ischemia models and endothelial experimental systems	[35]
Vascular smooth muscle cells	miR-143/145 cluster	Preserves the contractile phenotype and smooth muscle differentiation state	Smooth muscle differentiation and phenotype-switching regulatory networks	Phenotypic switching, increased motility, and vascular remodeling	Mouse knockout models, rat vascular injury models, and human aortic samples	[36]
Vascular smooth muscle cells	miR-21	Modulates smooth muscle cell proliferation, migration, and adaptive stress responses	Tropomyosin 1; HIF-1α/miR-21 axis	Enhanced proliferative remodeling and neointimal growth	Human arterial smooth muscle cells in arteriosclerosis-related settings	[37]
Vascular smooth muscle cells	miR-221/222	Promotes vascular cell plasticity and remodeling responses after injury	Networks associated with phenotype switching, proliferation, and motility	Transition toward a synthetic phenotype and progression of pathological vascular remodeling	Human vascular remodeling and atherosclerosis-related evidence	[38]
Cardiomyocytes	miR-1	Maintains electrical stability and contributes to excitation–contraction homeostasis	*GJA1* and *KCNJ2*	Arrhythmogenic susceptibility and disturbed cardiac conduction	Cardiac experimental models	[39]
Cardiomyocytes	miR-133	Supports cardiomyocyte homeostasis and limits maladaptive hypertrophic growth	*RHOA*, *CDC42*, and hypertrophy-related signaling programs	Loss of adaptive control of cardiomyocyte growth and increased remodeling susceptibility	Mouse and human hypertrophy-related models	[40]
Cardiomyocytes	miR-208 family	Regulates cardiac stress adaptation, myosin gene program, and myocardial identity	αMHC/βMHC-associated regulatory program	Hypertrophic remodeling, fibrosis, and altered myocardial performance under stress	Cardiac stress and hypothyroidism-related experimental models	[41]
Cardiac fibroblasts	miR-29a	Restrains fibroblast proliferation and extracellular matrix accumulation	Extracellular matrix regulatory networks	Enhanced fibroblast proliferation and profibrotic remodeling	Rat cardiac fibroblasts and fibrosis tissues	[42]
Cardiac fibroblasts	miR-21	Promotes fibroblast survival and profibrotic activation	ERK-MAPK signaling; *SPRY1*	Interstitial fibrosis, fibroblast activation, and cardiac dysfunction	Human, mouse, and rat cardiac fibrosis models	[43]

Abbreviations: ERK, extracellular signal-regulated kinase; HIF-1α, hypoxia-inducible factor 1 alpha; MAPK, mitogen-activated protein kinase; VCAM-1, vascular cell adhesion molecule 1; VEGF, vascular endothelial growth factor; VSMCs, vascular smooth muscle cells.

**Table 2:** MicroRNAs associated with specific cardiovascular diseases, reported expression, and clinical relevance.

Cardiovascular Disease	microRNA	Reported Expression Pattern	Main Process/Pathological Network Involved	Clinical Relevance	Sample/Source or Model	Reference
Coronary artery disease/atherosclerotic disease	miR-765	Upregulated	Atherosclerosis-associated vascular dysfunction	Candidate diagnostic biomarker	Human plasma, stable and unstable CAD cohorts	[142]
Coronary artery disease/atherosclerotic disease	miR-149	Downregulated	Atherosclerosis-associated regulatory networks	Candidate diagnostic biomarker	Human plasma, stable and unstable CAD cohorts	[142]
Hypertension	miR-27a	Downregulated	Blood pressure regulation and incident hypertension risk	Candidate predictive biomarker	Human serum, longitudinal population-based cohort	[143]
Hypertension	miR-133a	Downregulated	Blood pressure regulation and incident hypertension risk	Candidate predictive biomarker	Human serum, longitudinal population-based cohort	[143]
Hypertension with asymptomatic organ damage	miR-21	Upregulated	Vascular injury and remodeling-associated pathways	Candidate biomarker of subclinical target-organ damage	Human plasma, hypertensive patients	[144]
Acute myocardial infarction	miR-499	Upregulated	Cardiomyocyte injury and myocardial necrosis	Early diagnostic biomarker	Human plasma, AMI patients	[145]
Acute myocardial infarction	miR-19a	Upregulated	Myocardial injury-associated circulating signature	Candidate diagnostic biomarker	Human peripheral blood, AMI patients	[146]
Acute heart failure	miR-30d	Lower in non-survivors; higher levels associated with better survival	Heart failure-related stress and remodeling responses	Prognostic biomarker	Human serum, AHF cohort	[147]
Acute heart failure	miR-106a-5p	Downregulated	AHF severity, inflammatory burden, and poor prognosis	Diagnostic and prognostic biomarker	Human plasma, AHF cohort	[148]
Atrial fibrillation recurrence after catheter ablation	miR-15a-5p	Upregulated	Fibrosis-associated atrial remodeling	Predictive biomarker for recurrence	Human serum/exosomal samples, RFCA cohort	[149]
Early atrial fibrillation recurrence after catheter ablation	miR-206	Upregulated	Atrial remodeling associated with early recurrence	Predictive biomarker	Human plasma, prospective AF ablation cohort	[150]

Abbreviations: AF, atrial fibrillation; AHF, acute heart failure; AMI, acute myocardial infarction; CAD, coronary artery disease; RFCA, radiofrequency catheter ablation.

## Data Availability

No new data were created or analyzed in this study. Data sharing is not applicable to this article.

## Supplementary Materials

The following supporting information can be downloaded at https://www.mdpi.com/article/10.3390/ijms27083582/s1.

## Abbreviations

The following abbreviations are used in this manuscript:

ABCA1	ATP-binding cassette transporter A1
AF	atrial fibrillation
AHF	acute heart failure
ALDH2	aldehyde dehydrogenase 2
AMI	acute myocardial infarction
BNP	B-type natriuretic peptide
CAD	coronary artery disease
CMC	chemistry, manufacturing, and controls
DNA	deoxyribonucleic acid
ECM	extracellular matrix
EMA	European Medicines Agency
ERK	extracellular signal-regulated kinase
EVs	extracellular vesicles
FDA	Food and Drug Administration
HF	heart failure
HFpEF	heart failure with preserved ejection fraction
IL-1R	interleukin-1 receptor
IRAK1	interleukin-1 receptor-associated kinase 1
LNA	locked nucleic acid
MAPK	mitogen-activated protein kinase
miRNA	microRNA
MIQE	Minimum Information for Publication of Quantitative Real-Time PCR Experiments
MISEV2023	Minimal Information for Studies of Extracellular Vesicles 2023
NF-κB	nuclear factor kappa B
PDCD4	programmed cell death 4
PI3K	phosphoinositide 3-kinase
PTEN	phosphatase and tensin homolog
RFCA	radiofrequency catheter ablation
RISC	RNA-induced silencing complex
RNA	ribonucleic acid
ROS	reactive oxygen species
RT-qPCR	reverse transcription quantitative polymerase chain reaction
TGF-β	transforming growth factor beta
TLR	toll-like receptor
TRAF6	TNF receptor-associated factor 6
VCAM-1	vascular cell adhesion molecule 1
VEGF	vascular endothelial growth factor
VSMCs	vascular smooth muscle cells

## References

[1] Cardiovascular Diseases (CVDs). https://www.who.int/news-room/fact-sheets/detail/cardiovascular-diseases-(cvds).

[2] Bauersachs J., Thum T. (2011). Biogenesis and Regulation of Cardiovascular microRNAs. Circ. Res..

[3] O’Brien J., Hayder H., Zayed Y., Peng C. (2018). Overview of MicroRNA Biogenesis, Mechanisms of Actions, and Circulation. Front. Endocrinol..

[4] Searles C.D. (2024). MicroRNAs and Cardiovascular Disease Risk. Curr. Cardiol. Rep..

[5] Cordeiro M.A., de Carvalho A.E.T.S., Spadari R.C. (2025). MicroRNAs’ Impact on Heart Diseases. Int. J. Mol. Sci..

[6] Neppl R.L., Wang D.-Z. (2014). The Myriad Essential Roles of microRNAs in Cardiovascular Homeostasis and Disease. Genes Dis..

[7] Fernández-Hernando C., Suárez Y. (2018). MicroRNAs in Endothelial Cell Homeostasis and Vascular Disease. Curr. Opin. Hematol..

[8] Araldi E., Suárez Y. (2016). MicroRNAs as Regulators of Endothelial Cell Functions in Cardiometabolic Diseases. Biochim. Biophys. Acta.

[9] Wang S., Aurora A.B., Johnson B.A., Qi X., McAnally J., Hill J.A., Richardson J.A., Bassel-Duby R., Olson E.N. (2008). The Endothelial-Specific microRNA miR-126 Governs Vascular Integrity and Angiogenesis. Dev. Cell.

[10] Fish J.E., Santoro M.M., Morton S.U., Yu S., Yeh R.-F., Wythe J.D., Ivey K.N., Bruneau B.G., Stainier D.Y.R., Srivastava D. (2008). miR-126 Regulates Angiogenic Signaling and Vascular Integrity. Dev. Cell.

[11] Harris T.A., Yamakuchi M., Ferlito M., Mendell J.T., Lowenstein C.J. (2008). MicroRNA-126 Regulates Endothelial Expression of Vascular Cell Adhesion Molecule 1. Proc. Natl. Acad. Sci. USA.

[12] Kumar S., Kim C.W., Simmons R.D., Jo H. (2014). Role of Flow-Sensitive microRNAs in Endothelial Dysfunction and Atherosclerosis: Mechanosensitive Athero-miRs. Arterioscler. Thromb. Vasc. Biol..

[13] Silva V.R., Azar A., Goncalves E.R., Nascimento T.C.d.M., Buchaim R.L., Buchaim D.V., de Oliveira F.A.A., Nassar C.C., de Camargo T.M., Caboclo R.F. (2025). MicroRNA-Mediated Regulation of Vascular Endothelium: From Pro-Inflammation to Atherosclerosis. Int. J. Mol. Sci..

[14] Yan J., Zhong X., Zhao Y., Wang X. (2024). Role and Mechanism of miRNA in Cardiac Microvascular Endothelial Cells in Cardiovascular Diseases. Front. Cardiovasc. Med..

[15] Gupta S., Hernandez G., Raman P. (2025). Cellular and Molecular Mechanisms of VSMC Phenotypic Switching in Type 2 Diabetes. Cells.

[16] Khovantseva U., Markina Y., Kirichenko T., Goncharova K., Kiseleva D., Cherednichenko V., Markin A. (2025). Phenotypic Switching of VSMCs in the Development of CVDs: Focus on miRs. Int. J. Mol. Sci..

[17] Jang B., Zhang D., Ma Z., Yang X., Liu L., Xing H., Feng L., Song J., Zhao X., Song X. (2025). MicroRNAs in Vascular Smooth Muscle Cells: Mechanisms, Therapeutic Potential, and Advances in Delivery Systems. Life Sci..

[18] Cordes K.R., Sheehy N.T., White M., Berry E., Morton S.U., Muth A.N., Lee T.-H., Miano J.M., Ivey K.N., Srivastava D. (2009). miR-145 and miR-143 Regulate Smooth Muscle Cell Fate Decisions. Nature.

[19] Wang G., Luo Y., Gao X., Liang Y., Yang F., Wu J., Fang D., Luo M. (2023). MicroRNA Regulation of Phenotypic Transformations in Vascular Smooth Muscle: Relevance to Vascular Remodeling. Cell. Mol. Life Sci. CMLS.

[20] Liu X., Cheng Y., Zhang S., Lin Y., Yang J., Zhang C. (2009). A Necessary Role of miR-221 and miR-222 in Vascular Smooth Muscle Cell Proliferation and Neointimal Hyperplasia. Circ. Res..

[21] Brown S.D., Klimi E., Bakker W.A.M., Beqqali A., Baker A.H. (2025). Non-Coding RNAs to Treat Vascular Smooth Muscle Cell Dysfunction. Br. J. Pharmacol..

[22] Lozano-Velasco E., Inácio J.M., Sousa I., Guimarães A.R., Franco D., Moura G., Belo J.A. (2024). miRNAs in Heart Development and Disease. Int. J. Mol. Sci..

[23] Chen J.-F., Murchison E.P., Tang R., Callis T.E., Tatsuguchi M., Deng Z., Rojas M., Hammond S.M., Schneider M.D., Selzman C.H. (2008). Targeted Deletion of Dicer in the Heart Leads to Dilated Cardiomyopathy and Heart Failure. Proc. Natl. Acad. Sci. USA.

[24] Yang D., Deschênes I., Fu J.-D. (2022). Multilayer Control of Cardiac Electrophysiology by MicroRNAs. J. Mol. Cell. Cardiol..

[25] Huang X.-H., Li J.-L., Li X.-Y., Wang S.-X., Jiao Z.-H., Li S.-Q., Liu J., Ding J. (2021). miR-208a in Cardiac Hypertrophy and Remodeling. Front. Cardiovasc. Med..

[26] Yang D., Wan X., Schwieterman N., Cavus O., Kacira E., Xu X., Laurita K.R., Wold L.E., Hund T.J., Mohler P.J. (2024). MicroRNA-1 Deficiency Is a Primary Etiological Factor Disrupting Cardiac Contractility and Electrophysiological Homeostasis. Circ. Arrhythm. Electrophysiol..

[27] Yildiz M., Ozkan U., Budak M. (2025). The Heart’s Small Molecules: The Importance of MicroRNAs in Cardiovascular Health. J. Clin. Med..

[28] Shameem M., Olson S.L., Marron Fernandez de Velasco E., Kumar A., Singh B.N. (2025). Cardiac Fibroblasts: Helping or Hurting. Genes.

[29] Chimenti I., Pagano F., Cozzolino C., Icolaro F., Floris E., Picchio V. (2025). The Role of Cardiac Fibroblast Heterogeneity in Myocardial Fibrosis and Its Novel Therapeutic Potential. Int. J. Mol. Sci..

[30] Lin L.-C., Liu Z.-Y., Tu B., Song K., Sun H., Zhou Y., Sha J.-M., Zhang Y., Yang J.-J., Zhao J.-Y. (2024). Epigenetic Signatures in Cardiac Fibrosis: Focusing on Noncoding RNA Regulators as the Gatekeepers of Cardiac Fibroblast Identity. Int. J. Biol. Macromol..

[31] van Rooij E., Sutherland L.B., Thatcher J.E., DiMaio J.M., Naseem R.H., Marshall W.S., Hill J.A., Olson E.N. (2008). Dysregulation of microRNAs after Myocardial Infarction Reveals a Role of miR-29 in Cardiac Fibrosis. Proc. Natl. Acad. Sci. USA.

[32] Khalaji A., Mehrtabar S., Jabraeilipour A., Doustar N., Rahmani Youshanlouei H., Tahavvori A., Fattahi P., Alavi S.M.A., Taha S.R., Fazlollahpour-Naghibi A. (2024). Inhibitory Effect of microRNA-21 on Pathways and Mechanisms Involved in Cardiac Fibrosis Development. Ther. Adv. Cardiovasc. Dis..

[33] Cadosch N., Gil-Cruz C., Perez-Shibayama C., Ludewig B. (2024). Cardiac Fibroblastic Niches in Homeostasis and Inflammation. Circ. Res..

[34] Guo B., Gu J., Zhuang T., Zhang J., Fan C., Li Y., Zhao M., Chen R., Wang R., Kong Y. (2025). MicroRNA-126: From Biology to Therapeutics. Biomed. Pharmacother..

[35] Bonauer A., Carmona G., Iwasaki M., Mione M., Koyanagi M., Fischer A., Burchfield J., Fox H., Doebele C., Ohtani K. (2009). MicroRNA-92a Controls Angiogenesis and Functional Recovery of Ischemic Tissues in Mice. Science.

[36] Elia L., Quintavalle M., Zhang J., Contu R., Cossu L., Latronico M.V.G., Peterson K.L., Indolfi C., Catalucci D., Chen J. (2009). The Knockout of miR-143 and -145 Alters Smooth Muscle Cell Maintenance and Vascular Homeostasis in Mice: Correlates with Human Disease. Cell Death Differ..

[37] Wang M., Li W., Chang G.-Q., Ye C.-S., Ou J.-S., Li X.-X., Liu Y., Cheang T.-Y., Huang X.-L., Wang S.-M. (2011). MicroRNA-21 Regulates Vascular Smooth Muscle Cell Function via Targeting Tropomyosin 1 in Arteriosclerosis Obliterans of Lower Extremities. Arterioscler. Thromb. Vasc. Biol..

[38] Chistiakov D.A., Sobenin I.A., Orekhov A.N., Bobryshev Y.V. (2015). Human miR-221/222 in Physiological and Atherosclerotic Vascular Remodeling. BioMed Res. Int..

[39] Popat A., Jnaneswaran G., Yerukala Sathipati S., Sharma P.P. (2025). MicroRNAs in Cardiac Arrhythmias: Mechanisms, Biomarkers, and Therapeutic Frontiers. Heart Rhythm.

[40] Carè A., Catalucci D., Felicetti F., Bonci D., Addario A., Gallo P., Bang M.-L., Segnalini P., Gu Y., Dalton N.D. (2007). MicroRNA-133 Controls Cardiac Hypertrophy. Nat. Med..

[41] van Rooij E., Sutherland L.B., Qi X., Richardson J.A., Hill J., Olson E.N. (2007). Control of Stress-Dependent Cardiac Growth and Gene Expression by a microRNA. Science.

[42] Tao H., Chen Z.-W., Yang J.-J., Shi K.-H. (2016). MicroRNA-29a Suppresses Cardiac Fibroblasts Proliferation via Targeting VEGF-A/MAPK Signal Pathway. Int. J. Biol. Macromol..

[43] Thum T., Gross C., Fiedler J., Fischer T., Kissler S., Bussen M., Galuppo P., Just S., Rottbauer W., Frantz S. (2008). MicroRNA-21 Contributes to Myocardial Disease by Stimulating MAP Kinase Signalling in Fibroblasts. Nature.

[44] Nappi F., Avtaar Singh S.S., Jitendra V., Alzamil A., Schoell T. (2023). The Roles of microRNAs in the Cardiovascular System. Int. J. Mol. Sci..

[45] Zapata-Martínez L., Águila S., de Los Reyes-García A.M., Carrillo-Tornel S., Lozano M.L., González-Conejero R., Martínez C. (2023). Inflammatory microRNAs in Cardiovascular Pathology: Another Brick in the Wall. Front. Immunol..

[46] Abolhasani S., Ahmadi Y., Fattahi D., Rostami Y., Chollou K.M. (2025). microRNA-Mediated Regulation of Oxidative Stress in Cardiovascular Diseases. J. Clin. Lab. Anal..

[47] Gareev I., Beylerli O., Sufianov A., Gulieva L., Pavlov V., Shi H. (2025). MicroRNAs in the Regulation of Immune Response in Cardiovascular Diseases: New Diagnostic and Therapeutic Tools. Gene Expr..

[48] Mahdavi F.S., Mardi S., Mohammadi S., Ansari S., Yaslianifard S., Fallah P., Mozhgani S.-H. (2022). MicroRNA-146: Biomarker and Mediator of Cardiovascular Disease. Dis. Markers.

[49] Pauley K.M., Cha S., Chan E.K.L. (2009). MicroRNA in Autoimmunity and Autoimmune Diseases. J. Autoimmun..

[50] Sonkoly E., Pivarcsi A. (2009). Advances in microRNAs: Implications for Immunity and Inflammatory Diseases. J. Cell. Mol. Med..

[51] Mezzetti E., Costantino A., Leoni M., Pieretti R., Di Paolo M., Frati P., Maiese A., Fineschi V. (2023). Autoimmune Heart Disease: A Comprehensive Summary for Forensic Practice. Medicina.

[52] Grodzka O., Procyk G., Gąsecka A. (2022). The Role of MicroRNAs in Myocarditis-What Can We Learn from Clinical Trials?. Int. J. Mol. Sci..

[53] Liu W., Hu J., Lu S., Wang Z. (2022). The Role of Non-Coding RNAs in Myocarditis: A Narrative Review. Ann. Transl. Med..

[54] Iside C., Picone F., Di Pietro P., Abate A.C., Prete V., Damato A., Venturini E., Akeel S., Petralia S., Vecchione C. (2025). MicroRNA Signatures in Cardiometabolic Disorders as a Next-Generation Diagnostic Approach: Current Insight. Int. J. Mol. Sci..

[55] Wang J., Li Y., Wang H., Meng Q., Li P., Wang Y., Wang K., Yang S. (2025). Harnessing miRNA Therapeutics: A Novel Approach to Combat Heart and Brain Infarctions in Atherosclerosis. Cell Death Discov..

[56] Yang H.-M. (2025). Mitochondrial Dysfunction in Cardiovascular Diseases. Int. J. Mol. Sci..

[57] Xiang M., Yang M., Zhang L., Ouyang X., Sarapultsev A., Luo S., Hu D. (2025). Mitochondrial DNA Dysfunction in Cardiovascular Diseases: A Novel Therapeutic Target. Antioxidants.

[58] Quiroga D., Daniel R., Das S. (2025). Mechanisms of microRNA Trafficking to Mitochondria in the Heart. Curr. Opin. Physiol..

[59] Song R., Dasgupta C., Mulder C., Zhang L. (2022). MicroRNA-210 Controls Mitochondrial Metabolism and Protects Heart Function in Myocardial Infarction. Circulation.

[60] Banavath H.N., Roman B., Mackowski N., Biswas D., Afzal J., Nomura Y., Solhjoo S., O’Rourke B., Kohr M., Murphy E. (2019). miR-181c Activates Mitochondrial Calcium Uptake by Regulating MICU1 in the Heart. J. Am. Heart Assoc..

[61] Kaur S., Bhatti G.K., Khullar N., Bhatti J.S. (2026). Targeting Mitochondrial microRNAs in Cardiovascular Pathologies: A New Frontier in Precision Cardiology. Ageing Res. Rev..

[62] Méndez-García A., García-Mendoza M.A., Zárate-Peralta C.P., Flores-Perez F.V., Carmona-Ramirez L.F., Pathak S., Banerjee A., Duttaroy A.K., Paul S. (2025). Mitochondrial microRNAs (mitomiRs) as Emerging Biomarkers and Therapeutic Targets for Chronic Human Diseases. Front. Genet..

[63] Kansakar U., Varzideh F., Mone P., Jankauskas S.S., Santulli G. (2022). Functional Role of microRNAs in Regulating Cardiomyocyte Death. Cells.

[64] Mohammed O.A., Alghamdi M., Alfaifi J., Alamri M.M.S., Al-Shahrani A.M., Alharthi M.H., Alshahrani A.M., Alhalafi A.H., Adam M.I.E., Bahashwan E. (2024). The Emerging Role of miRNAs in Myocardial Infarction: From Molecular Signatures to Therapeutic Targets. Pathol. Res. Pract..

[65] Cheng Y., Liu X., Zhang S., Lin Y., Yang J., Zhang C. (2009). MicroRNA-21 Protects against the H_2_O_2_-Induced Injury on Cardiac Myocytes via Its Target Gene PDCD4. J. Mol. Cell. Cardiol..

[66] Qian L., Van Laake L.W., Huang Y., Liu S., Wendland M.F., Srivastava D. (2011). miR-24 Inhibits Apoptosis and Represses Bim in Mouse Cardiomyocytes. J. Exp. Med..

[67] Boon R.A., Iekushi K., Lechner S., Seeger T., Fischer A., Heydt S., Kaluza D., Tréguer K., Carmona G., Bonauer A. (2013). MicroRNA-34a Regulates Cardiac Ageing and Function. Nature.

[68] Liu N., Zhen Z., Xiong X., Xue Y. (2024). Aerobic Exercise Protects MI Heart through miR-133a-3p Downregulation of Connective Tissue Growth Factor. PLoS ONE.

[69] Fumarulo I., De Prisco A., Salerno E.N.M., Ravenna S.E., Vaccarella M., Garramone B., Burzotta F., Aspromonte N. (2025). New Frontiers of microRNA in Heart Failure: From Clinical Risk to Therapeutic Applications. J. Clin. Med..

[70] Ghazal R., Wang M., Liu D., Tschumperlin D.J., Pereira N.L. (2025). Cardiac Fibrosis in the Multi-Omics Era: Implications for Heart Failure. Circ. Res..

[71] Gocer Z., Elek A., Caska H., Bozgeyik I. (2023). MicroRNAs and Cardiac Fibrosis: A Comprehensive Update on Mechanisms and Consequences. Pathol. Res. Pract..

[72] Pan Z., Sun X., Shan H., Wang N., Wang J., Ren J., Feng S., Xie L., Lu C., Yuan Y. (2012). MicroRNA-101 Inhibited Postinfarct Cardiac Fibrosis and Improved Left Ventricular Compliance via the FBJ Osteosarcoma Oncogene/Transforming Growth Factor-Β1 Pathway. Circulation.

[73] Micu M.A., Cozac D.A., Scridon A. (2025). miRNA-Orchestrated Fibroinflammatory Responses in Heart Failure with Preserved Ejection Fraction: Translational Opportunities for Precision Medicine. Diagnostics.

[74] Laggerbauer B., Engelhardt S. (2022). MicroRNAs as Therapeutic Targets in Cardiovascular Disease. J. Clin. Investig..

[75] D’Amato A., Prosperi S., Severino P., Myftari V., Correale M., Perrone Filardi P., Badagliacca R., Fedele F., Vizza C.D., Palazzuoli A. (2024). MicroRNA and Heart Failure: A Novel Promising Diagnostic and Therapeutic Tool. J. Clin. Med..

[76] Solly E.L., Dimasi C.G., Bursill C.A., Psaltis P.J., Tan J.T.M. (2019). MicroRNAs as Therapeutic Targets and Clinical Biomarkers in Atherosclerosis. J. Clin. Med..

[77] Montgomery R.L., Hullinger T.G., Semus H.M., Dickinson B.A., Seto A.G., Lynch J.M., Stack C., Latimer P.A., Olson E.N., van Rooij E. (2011). Therapeutic Inhibition of miR-208a Improves Cardiac Function and Survival During Heart Failure. Circulation.

[78] Shang Z., Huang K. (2025). Cyclic Stretch and Oxidative Stress Induced miRNAs in Vascular Remodeling. Biophys. Rep..

[79] Gilyazova I., Timasheva Y., Chumakova A., Abdeeva G., Plotnikova M., Zagidullin N. (2025). The Role of MicroRNAs in the Pathophysiology and Management of Heart Failure: From Molecular Mechanisms to Clinical Application. Int. J. Mol. Sci..

[80] Gong S., Li Y., Yan K., Shi Z., Leng J., Bao Y., Ning K. (2025). The Crosstalk Between Endothelial Cells, Smooth Muscle Cells, and Macrophages in Atherosclerosis. Int. J. Mol. Sci..

[81] Zaidi S.A., Fan Z., Chauhdari T., Ding Y. (2025). MicroRNA Regulatory Dynamic, Emerging Diagnostic and Therapeutic Frontier in Atherosclerosis. Microvasc. Res..

[82] Rayner K.J., Sheedy F.J., Esau C.C., Hussain F.N., Temel R.E., Parathath S., van Gils J.M., Rayner A.J., Chang A.N., Suarez Y. (2011). Antagonism of miR-33 in Mice Promotes Reverse Cholesterol Transport and Regression of Atherosclerosis. J. Clin. Investig..

[83] Ouimet M., Ediriweera H., Afonso M.S., Ramkhelawon B., Singaravelu R., Liao X., Bandler R.C., Rahman K., Fisher E.A., Rayner K.J. (2017). microRNA-33 Regulates Macrophage Autophagy in Atherosclerosis. Arterioscler. Thromb. Vasc. Biol..

[84] Nazari-Jahantigh M., Wei Y., Noels H., Akhtar S., Zhou Z., Koenen R.R., Heyll K., Gremse F., Kiessling F., Grommes J. (2012). MicroRNA-155 Promotes Atherosclerosis by Repressing Bcl6 in Macrophages. J. Clin. Investig..

[85] Sič A., Atanasković M., Ahmed A., Petrović I., Simović F., Burnjaković B., Tonković U., Manzar A., Shadab S., Gajić S. (2025). The Association of MicroRNA-21 with Carotid Artery Disease and Ischemic Stroke: From Pathophysiology to Clinical Implications and Potential Therapy. Med. Sci..

[86] Wong Z.W., Thanikachalam P.V., Ramamurthy S. (2026). Emerging Insights into MicroRNA Therapeutics and Diagnostics for Atherosclerotic Cardiovascular Disease: A Narrative Review. Int. J. Biol. Macromol..

[87] He L., Liu Y., Widlansky M.E., Kriegel A.J., Qiu Q., Liang M. (2025). microRNA and Hypertension. Hypertension.

[88] Adamcova M., Kawano I., Simko F. (2021). The Impact of microRNAs in Renin–Angiotensin-System-Induced Cardiac Remodelling. Int. J. Mol. Sci..

[89] Nemecz M., Alexandru N., Tanko G., Georgescu A. (2016). Role of MicroRNA in Endothelial Dysfunction and Hypertension. Curr. Hypertens. Rep..

[90] Wang C., Wu H., Xing Y., Ye Y., He F., Yin Q., Li Y., Shang F., Shyy J.Y.-J., Yuan Z.-Y. (2022). Endothelial-Derived Extracellular microRNA-92a Promotes Arterial Stiffness by Regulating Phenotype Changes of Vascular Smooth Muscle Cells. Sci. Rep..

[91] Marques F.Z., Romaine S.P., Denniff M., Eales J., Dormer J., Garrelds I.M., Wojnar L., Musialik K., Duda-Raszewska B., Kiszka B. (2015). Signatures of miR-181a on the Renal Transcriptome and Blood Pressure. Mol. Med..

[92] Watanabe K., Narumi T., Watanabe T., Otaki Y., Takahashi T., Aono T., Goto J., Toshima T., Sugai T., Wanezaki M. (2020). The Association between microRNA-21 and Hypertension-Induced Cardiac Remodeling. PLoS ONE.

[93] Sun H.-X., Zeng D.-Y., Li R.-T., Pang R.-P., Yang H., Hu Y.-L., Zhang Q., Jiang Y., Huang L.-Y., Tang Y.-B. (2012). Essential Role of MicroRNA-155 in Regulating Endothelium-Dependent Vasorelaxation by Targeting Endothelial Nitric Oxide Synthase. Hypertension.

[94] Kostiniuk D., Marttila S., Raitoharju E. (2025). Circulatory miRNAs in Essential Hypertension. Atherosclerosis.

[95] Reel S., Reel P.S., Van Kralingen J., Larsen C.K., Robertson S., MacKenzie S.M., Riddell A., McClure J.D., Lamprou S., Connell J.M.C. (2025). Identification of Hypertension Subtypes Using microRNA Profiles and Machine Learning. Eur. J. Endocrinol..

[96] Zhu M., Li Y., Xu Q., Wang W., Liu Y., Liu Y. (2025). Acute Myocardial Infarction: Molecular Pathogenesis, Diagnosis, and Clinical Management. MedComm.

[97] Wang G.-K., Zhu J.-Q., Zhang J.-T., Li Q., Li Y., He J., Qin Y.-W., Jing Q. (2010). Circulating microRNA: A Novel Potential Biomarker for Early Diagnosis of Acute Myocardial Infarction in Humans. Eur. Heart J..

[98] Rastegar-Moghaddam S.H., Bigham M., Lombardi G., Mohammadipour A., Malvandi A.M. (2024). MicroRNA-24 Therapeutic Potentials in Infarction, Stroke, and Diabetic Complications. Mol. Biol. Rep..

[99] Burnjaković B., Atanasković M., Baralić M., Altić A., Nikolov E., Ilić A., Sič A., Stanković Popović V., Bontić A., Gajić S. (2026). Diagnostic, Prognostic and Therapeutic Utility of MicroRNA-21 in Ischemic Heart Disease. Int. J. Mol. Sci..

[100] Armbruster A.L., Campbell K.B., Kahanda M.G., Cuculich P.S. (2022). The Role of Inflammation in the Pathogenesis and Treatment of Arrhythmias. Pharmacotherapy.

[101] Dobrev D., Heijman J., Hiram R., Li N., Nattel S. (2023). Inflammatory Signalling in Atrial Cardiomyocytes: A Novel Unifying Principle in Atrial Fibrillation Pathophysiology. Nat. Rev. Cardiol..

[102] Hu J., Huang C.-X., Rao P.-P., Zhou J.-P., Wang X., Tang L., Liu M.-X., Zhang G.-G. (2019). Inhibition of microRNA-155 Attenuates Sympathetic Neural Remodeling Following Myocardial Infarction via Reducing M1 Macrophage Polarization and Inflammatory Responses in Mice. Eur. J. Pharmacol..

[103] Khizar M., Zaib M., Bilal M., Aminpoor H., Karimi H. (2025). Circulating microRNAs in Post Myocardial Infarction Remodeling: Toward Precision Cardiology. Ann. Med. Surg..

[104] Song R., Zhang L. (2024). MicroRNAs and Therapeutic Potentials in Acute and Chronic Cardiac Disease. Drug Discov. Today.

[105] Gargiulo P., Marzano F., Salvatore M., Basile C., Buonocore D., Parlati A.L.M., Nardi E., Asile G., Abbate V., Colella A. (2023). MicroRNAs: Diagnostic, Prognostic and Therapeutic Role in Heart Failure—A Review. ESC Heart Fail..

[106] Profire B.-Ș., Lupașcu F.G., Stătescu C., Șorodoc V., Sascău R.-A., Profire L., Șorodoc L. (2025). Heart Failure Biomarkers—Pathophysiology, Diagnosis, Prognosis and Clinical Relevance. Int. J. Mol. Sci..

[107] Parvan R., Becker V., Hosseinpour M., Moradi Y., Louch W.E., Cataliotti A., Devaux Y., Frisk M., Silva G.J.J. (2025). Prognostic and Predictive microRNA Panels for Heart Failure Patients with Reduced or Preserved Ejection Fraction: A Meta-Analysis of Kaplan–Meier-Based Individual Patient Data. BMC Med..

[108] Guo X., Zhou Y., Huang H., Zong Z., Xin M., Yang K. (2024). Diagnostic and Prognostic Value of microRNA423-5p in Patients with Heart Failure. J. Cardiothorac. Surg..

[109] Kattih B., Fischer A., Muhly-Reinholz M., Tombor L., Nicin L., Cremer S., Zeiher A.M., John D., Abplanalp W.T., Dimmeler S. (2025). Inhibition of miR-92a Normalizes Vascular Gene Expression and Prevents Diastolic Dysfunction in Heart Failure with Preserved Ejection Fraction. J. Mol. Cell. Cardiol..

[110] Țica O., Țica O. (2025). Molecular Diagnostics in Heart Failure: From Biomarkers to Personalized Medicine. Diagnostics.

[111] Balan A.I., Scridon A. (2025). MicroRNAs in Atrial Fibrillation—Have We Discovered the Holy Grail or Opened a Pandora’s Box?. Front. Pharmacol..

[112] Wiedmann F., Kraft M., Kallenberger S., Büscher A., Paasche A., Blochberger P.L., Seeger T., Jávorszky N., Warnecke G., Arif R. (2022). MicroRNAs Regulate TASK-1 and Are Linked to Myocardial Dilatation in Atrial Fibrillation. J. Am. Heart Assoc..

[113] Balan A.I., Halaţiu V.B., Comșulea E., Mutu C.C., Cozac D.A., Aspru I., Păcurar D., Bănescu C., Perian M., Scridon A. (2025). The Diagnostic and Predictive Potential of miR-328 in Atrial Fibrillation: Insights from a Spontaneously Hypertensive Rat Model. Int. J. Mol. Sci..

[114] Adam O., Löhfelm B., Thum T., Gupta S.K., Puhl S.-L., Schäfers H.-J., Böhm M., Laufs U. (2012). Role of miR-21 in the Pathogenesis of Atrial Fibrosis. Basic Res. Cardiol..

[115] Vardas E.P., Oikonomou E., Vardas P.E., Tousoulis D. (2024). MicroRNAs as Prognostic Biomarkers for Atrial Fibrillation Recurrence After Catheter Ablation: Current Evidence and Future Directions. Biomedicines.

[116] Wang J., Wang Y., Han J., Li Y., Xie C., Xie L., Shi J., Zhang J., Yang B., Chen D. (2015). Integrated Analysis of microRNA and mRNA Expression Profiles in the Left Atrium of Patients with Nonvalvular Paroxysmal Atrial Fibrillation: Role of miR-146b-5p in Atrial Fibrosis. Heart Rhythm.

[117] Tak K., Lessard D., Kiefe C.I., Freedman J.E., Parker M., Aurigemma G.P., Donahue K., McManus D.D., Tran K.-V. (2025). Circulating Extra-Cellular RNAs and Atrial Fibrillation: Data from the TRACE-CORE Cohort. Front. Cardiovasc. Med..

[118] Yerukala Sathipati S., Carter T., Soodi D., Somto N., Shukla S.K., Petronovich J., Ingrid G., Braxton J., Sharma P. (2025). MicroRNA Signature Predicts Post Operative Atrial Fibrillation after Coronary Artery Bypass Grafting. Sci. Rep..

[119] Wang H.-T., Chen S.-M., Chen H.-C., Lin P.-T., Chen Y.-L. (2025). miR-1-3p Downregulation as a Consistent Biomarker for Atrial Fibrillation Burden in Patients with Sick Sinus Syndrome: A Multi-Sample Analysis. Int. J. Mol. Sci..

[120] Sacco M.A., Gualtieri S., Verrina M.C., Cordasco F., Monterossi M.D., Grimaldi G., Mastrangelo H., Mazza G., Aquila I. (2026). MicroRNAs in Cardiovascular Diseases and Forensic Applications: A Systematic Review of Diagnostic and Post-Mortem Implications. Int. J. Mol. Sci..

[121] Katayama E.S., Hue J.J., Loftus A.W., Ali S.A., Graor H.J., Rothermel L.D., Londin E., Zarei M., Winter J.M. (2025). Stability of microRNAs in Serum and Plasma Reveal Promise as a Circulating Biomarker. Non-Coding RNA Res..

[122] Felekkis K., Papaneophytou C. (2020). Challenges in Using Circulating Micro-RNAs as Biomarkers for Cardiovascular Diseases. Int. J. Mol. Sci..

[123] Alcibahy Y., Darwish R., Abu-Sharia G., Maes Q., Elgamassy O. (2025). Circulating microRNAs as Biomarkers for Ischemic Heart Disease: A Systematic Review and Gene Set Enrichment Analysis. Front. Med..

[124] Stojkovic S., Wojta J. (2024). Circulatory microRNAs in Acute Coronary Syndrome: An Update. Kardiol. Pol..

[125] Zendjabil M. (2024). Preanalytical, Analytical and Postanalytical Considerations in Circulating microRNAs Measurement. Biochem. Medica.

[126] Pastor-Navarro B., Ramírez-Calvo M., Gil-Aldea I., Cortell-Granero I., López-Guerrero J.A. (2024). The Impact of Tube Type, Centrifugation Conditions, and Hemolysis on Plasma Circulating MicroRNAs. Diagnostics.

[127] Cappucci I.P., Tremoli E., Zavan B., Ferroni L. (2025). Circulating Extracellular Vesicles in Cardiovascular Disease. Int. J. Mol. Sci..

[128] Li T., Wang W., Qin Z., Chen Y., Zhu K., Liu H., Sun J., Zhong H. (2025). Extracellular Vesicles in Cardiovascular Diseases: Pathogenic Mediators, Diagnostic Tools, and Therapeutic Vectors. Front. Cardiovasc. Med..

[129] Limpitikul W.B., Silverman M.G., Valkov N., Park J.-G., Yeri A., Garcia F.C., Li G., Gokulnath P., Garcia-Contreras M., Alsop E. (2025). Plasma Extracellular Vesicle Cargo microRNAs Are Associated with Heart Failure and Cardiovascular Death Following Acute Coronary Syndrome. Extracell. Vesicle.

[130] Bernáth-Nagy D., Kalinyaprak M.S., Giannitsis E., Ábrahám P., Leuschner F., Frey N., Krohn J.B. (2024). Circulating Extracellular Vesicles as Biomarkers in the Diagnosis, Prognosis and Therapy of Cardiovascular Diseases. Front. Cardiovasc. Med..

[131] Chae C.-W., Choi G., Yoon T., Kwon Y.-W. (2025). Exosome-Based Therapy in Cardiovascular Diseases: A New Frontier in Cardiovascular Disease Treatment. Korean Circ. J..

[132] Chen M., Wu Y., Chen C. (2025). Extracellular Vesicles as Emerging Regulators in Ischemic and Hypertrophic Cardiovascular Diseases: A Review of Pathogenesis and Therapeutics. Med. Sci. Monit..

[133] Wang Y., Yuan Y., Tang J. (2025). Extracellular Vesicles as Diagnostic Metrics for Cardiovascular Disease: Where We Are and How to Achieve in Clinics. J Cardiovasc. Transl. Res..

[134] Nazir A., Nazir A., Afzaal U., Aman S., Sadiq S.U.R., Akah O.Z., Jamal M.S.W., Hassan S.Z. (2025). Advancements in Biomarkers for Early Detection and Risk Stratification of Cardiovascular Diseases—A Literature Review. Health Sci. Rep..

[135] Vafadar A., Alavimanesh S., Babadi S., Hosseinpour V., Ehtiati S., Savardashtaki A. (2026). MicroRNA Biosensors for Cardiovascular Disease Detection. Clin. Chim. Acta.

[136] Reich C., Kayvanpour E., Sedaghat-Hamedani F., Amr A., Haas J., Ueltzhöffer K., Plebani M., Padoan A., Lind L., Lindahl B. (2025). The European BestAgeing Study on microRNA Candidates Reveals Distinct Signatures with Diagnostic and Prognostic Potential in Cardiovascular Disease. BMC Med..

[137] Vasu M.M., Linda K., Sanjay G., Jeemon P., Urulangodi M., Gopala S., Harikrishnan S. (2025). Diagnostic and Prognostic Relevance of Circulating MicroRNAs in Heart Failure. Heart Fail. J. India.

[138] Rodríguez-Serrano M., Martín-García E., Alonso-Andrés P., Conde-Moreno E., Pian H., del Moral-Salmoral J., Alcharani N., Menacho-Román M., Crespo-Toro L., Ramos-Muñoz M.E. (2026). Dynamic microRNA Signatures as Biomarkers for Cardiac Ischemia and Remodeling. Int. J. Mol. Sci..

[139] Shirakabe A., Ikeda Y., Uchikado Y., Okazaki H., Matsushita M., Sawatani T., Shigihara S., Tani K., Morooka M., Takahashi M. (2025). Prognostic Impact of Mitochondrial Dynamics-Related miRNA Levels during the Treatment of Acute Heart Failure in the Hospital. Sci. Rep..

[140] Bokhari S.F.H., Umais M., Faizan Sattar S.M., Mehboob U., Iqbal A., Amir M., Bakht D., Ali K., Hasan A.H., Javed M.A. (2025). Novel Cardiac Biomarkers and Multiple-Marker Approach in the Early Detection, Prognosis, and Risk Stratification of Cardiac Diseases. World J. Cardiol..

[141] Ngo T.H., Tran S.K. (2025). Role of Polymorphisms and microRNA Levels in Predicting Cardiovascular Events in Patients with Acute Myocardial Infarction. World J. Cardiol..

[142] Ali Sheikh M.S., Xia K., Li F., Deng X., Salma U., Deng H., Wei Wei L., Yang T.-L., Peng J. (2015). Circulating miR-765 and miR-149: Potential Noninvasive Diagnostic Biomarkers for Geriatric Coronary Artery Disease Patients. BioMed Res. Int..

[143] Suzuki K., Yamada H., Fujii R., Munetsuna E., Yamazaki M., Ando Y., Ohashi K., Ishikawa H., Mizuno G., Tsuboi Y. (2022). Circulating microRNA-27a and -133a Are Negatively Associated with Incident Hypertension: A Five-Year Longitudinal Population-Based Study. Biomarkers.

[144] Yildirim E., Ermis E., Allahverdiyev S., Ucar H., Yavuzer S., Cengiz M. (2019). Circulating miR-21 Levels in Hypertensive Patients with Asymptomatic Organ Damage. Medicine.

[145] Adachi T., Nakanishi M., Otsuka Y., Nishimura K., Hirokawa G., Goto Y., Nonogi H., Iwai N. (2010). Plasma microRNA 499 as a Biomarker of Acute Myocardial Infarction. Clin. Chem..

[146] Zhong J., He Y., Chen W., Shui X., Chen C., Lei W. (2014). Circulating microRNA-19a as a Potential Novel Biomarker for Diagnosis of Acute Myocardial Infarction. Int. J. Mol. Sci..

[147] Xiao J., Gao R., Bei Y., Zhou Q., Zhou Y., Zhang H., Jin M., Wei S., Wang K., Xu X. (2017). Circulating miR-30d Predicts Survival in Patients with Acute Heart Failure. Cell. Physiol. Biochem..

[148] Fei A., Li L., Li Y., Zhou T., Liu Y. (2024). Diagnostic and Prognostic Value of Plasma miR-106a-5p Levels in Patients with Acute Heart Failure. J. Cardiothorac. Surg..

[149] Ruan Z.-B., Peng Q., Sun X.-J., Wang F., Zhu L. (2025). Serum miR-15a-5p May Predict Recurrence of Atrial Fibrillation after Catheter Ablation: A Single Center Retrospective Study. PeerJ.

[150] Šustr F., Macháčková T., Pešl M., Svačinova J., Trachtová K., Stárek Z., Kianička B., Slabý O., Novák J. (2024). Identification of Plasmatic MicroRNA-206 as New Predictor of Early Recurrence of Atrial Fibrillation After Catheter Ablation Using Next-Generation Sequencing. Mol. Diagn. Ther..

[151] Dzau V.J., Hodgkinson C.P. (2024). RNA Therapeutics for the Cardiovascular System. Circulation.

[152] Dui W., Xiaobin Z., Haifeng Z., Lijuan D., Wenhui H., Zhengfeng Z., Jinling S. (2025). Harnessing RNA Therapeutics: Novel Approaches and Emerging Strategies for Cardiovascular Disease Management. Front. Cardiovasc. Med..

[153] Wang Z., Peng Y., Zhou H., Zhang M., Ju D., Chen Z. (2025). MiRNA-Based Drugs: Challenges and Delivery Strategies. Appl. Microbiol. Biotechnol..

[154] Hernández-Bellido N., Hernández-Ainsa S., Köhler R., Ordovás L. (2026). Nanotechnology-Driven Heart Targeting to Fulfil the Potential of Cardiac microRNA Therapies. Int. J. Pharm..

[155] Brillante S., Volpe M., Indrieri A. (2024). Advances in MicroRNA Therapeutics: From Preclinical to Clinical Studies. Hum. Gene Ther..

[156] Bauersachs J., Solomon S.D., Anker S.D., Antorrena-Miranda I., Batkai S., Viereck J., Rump S., Filippatos G., Granzer U., Ponikowski P. (2024). Efficacy and Safety of CDR132L in Patients with Reduced Left Ventricular Ejection Fraction After Myocardial Infarction: Rationale and Design of the HF-REVERT Trial. Eur. J. Heart Fail..

[157] Täubel J., Hauke W., Rump S., Viereck J., Batkai S., Poetzsch J., Rode L., Weigt H., Genschel C., Lorch U. (2021). Novel Antisense Therapy Targeting microRNA-132 in Patients with Heart Failure: Results of a First-in-Human Phase 1b Randomized, Double-Blind, Placebo-Controlled Study. Eur. Heart J..

[158] Peters L.J.F., Bidzhekov K., Bonnin-Marquez A., Sundararaman S.S., Huchzermeier R., Maas S.L., Abschlag K., Jans A., Lin C., Haberbosch M. (2025). MicroRNA-26b-/- Augments Atherosclerosis, While Mimic-Loaded Nanoparticles Reduce Atherogenesis. Cardiovasc. Res..

[159] Olianti C., Giacca M. (2025). Proregenerative MicroRNAs to Repair the Damaged Heart. Eur. Cardiol. Rev..

[160] Saenz-Pipaon G., Dichek D.A. (2023). Targeting and Delivery of microRNA-Targeting Antisense Oligonucleotides in Cardiovascular Diseases. Atherosclerosis.

[161] Horie T., Baba O., Nishino T., Yamashita Y., Tsujisaka Y., Ono K. (2026). Critical Role of microRNA-33a/b in Cardiovascular and Metabolic Disease: Molecular Mechanisms and Therapeutic Perspectives. J. Atheroscler. Thromb..

[162] Abplanalp W.T., Fischer A., John D., Zeiher A.M., Gosgnach W., Darville H., Montgomery R., Pestano L., Allée G., Paty I. (2020). Efficiency and Target Derepression of Anti-miR-92a: Results of a First in Human Study. Nucleic Acid Ther..

[163] Ruan H., Dou D., Lu J., Xiao X., Gong X., Zhang X. (2025). Off-Target Effects of Oligonucleotides and Approaches of Preclinical Assessments. SLAS Discov..

[164] Bereczki Z., Benczik B., Balogh O.M., Marton S., Puhl E., Pétervári M., Váczy-Földi M., Papp Z.T., Makkos A., Glass K. (2025). Mitigating Off-Target Effects of Small RNAs: Conventional Approaches, Network Theory and Artificial Intelligence. Br. J. Pharmacol..

[165] Nicoletti L., Coletto M., Stola G.P., Paoletti C., Marcello E., Testore D., Tivano F., Zoso A., Mattu C., Chiono V. (2025). Strategies for the Delivery of RNA Therapeutics to Diseased Heart Tissue. Expert Opin. Drug Deliv..

[166] Wang N., Chen C., Ren J., Dai D. (2024). MicroRNA Delivery Based on Nanoparticles of Cardiovascular Diseases. Mol. Cell. Biochem..

[167] Bheri S., Davis M.E. (2019). Nanoparticle-Hydrogel System for Post-Myocardial Infarction Delivery of MicroRNA. ACS Nano.

[168] Bejerano T., Etzion S., Elyagon S., Etzion Y., Cohen S. (2018). Nanoparticle Delivery of miRNA-21 Mimic to Cardiac Macrophages Improves Myocardial Remodeling after Myocardial Infarction. Nano Lett..

[169] Sun B., Liu S., Hao R., Dong X., Fu L., Han B. (2020). RGD-PEG-PLA Delivers MiR-133 to Infarct Lesions of Acute Myocardial Infarction Model Rats for Cardiac Protection. Pharmaceutics.

[170] Chen Y., Liu S., Liang Y., He Y., Li Q., Zhan J., Hou H., Qiu X. (2024). Single Dose of Intravenous miR199a-5p Delivery Targeting Ischemic Heart for Long-Term Repair of Myocardial Infarction. Nat. Commun..

[171] de Abreu R.C., Fernandes H., da Costa Martins P.A., Sahoo S., Emanueli C., Ferreira L. (2020). Native and Bioengineered Extracellular Vesicles for Cardiovascular Therapeutics. Nat. Rev. Cardiol..

[172] Wang R., Yi L., Zhou W., Wang W., Wang L., Xu L., Deng C., He M., Xie Y., Xu J. (2023). Targeted microRNA Delivery by Lipid Nanoparticles and Gas Vesicle-Assisted Ultrasound Cavitation to Treat Heart Transplant Rejection. Biomater. Sci..

[173] Seyhan A.A. (2024). Trials and Tribulations of MicroRNA Therapeutics. Int. J. Mol. Sci..

[174] Allen R., Yokota T. (2024). Endosomal Escape and Nuclear Localization: Critical Barriers for Therapeutic Nucleic Acids. Molecules.

[175] Gil-Cabrerizo P., Simon-Yarza T., Garbayo E., Blanco-Prieto M.J. (2024). Navigating the Landscape of RNA Delivery Systems in Cardiovascular Disease Therapeutics. Adv. Drug Deliv. Rev..

[176] Hong D.S., Kang Y.-K., Borad M., Sachdev J., Ejadi S., Lim H.Y., Brenner A.J., Park K., Lee J.-L., Kim T.-Y. (2020). Phase 1 Study of MRX34, a Liposomal miR-34a Mimic, in Patients with Advanced Solid Tumours. Br. J. Cancer.

[177] Center for Drug Evaluation and Research Drug Products, Including Biological Products, That Contain Nanomaterials—Guidance for Industry. https://www.fda.gov/regulatory-information/search-fda-guidance-documents/drug-products-including-biological-products-contain-nanomaterials-guidance-industry.

[178] Development and Manufacture of Oligonucleotides—Scientific Guideline|European Medicines Agency (EMA). https://www.ema.europa.eu/en/development-manufacture-oligonucleotides-scientific-guideline.

[179] Center for Drug Evaluation and Research Clinical Pharmacology Considerations for the Development of Oligonucleotide Therapeutics. https://www.fda.gov/regulatory-information/search-fda-guidance-documents/clinical-pharmacology-considerations-development-oligonucleotide-therapeutics.

[180] Bustin S.A., Ruijter J.M., van den Hoff M.J.B., Kubista M., Pfaffl M.W., Shipley G.L., Tran N., Rödiger S., Untergasser A., Mueller R. (2025). MIQE 2.0: Revision of the Minimum Information for Publication of Quantitative Real-Time PCR Experiments Guidelines. Clin. Chem..

[181] Drude N., Baselly C., Gazda M.A., May J.-N., Tienken L., Abbasi P., Weissgerber T., Burgess S. (2026). Reporting Quality of Quantitative Polymerase Chain Reaction (qPCR) Methods in Scientific Publications. Res. Integr. Peer Rev..

[182] Samuels M., Giamas G. (2024). MISEV2023: Shaping the Future of EV Research by Enhancing Rigour, Reproducibility and Transparency. Cancer Gene Ther..

[183] Li K., Samiei S., Pikulska D., Foecking S., Kuppe C. (2026). Advancing Cardiovascular Research with Single-Cell and Spatial Transcriptomics. Circ. Res..

[184] Kreutz J., Mitić T., Caporali A. (2025). Exploring microRNAs, One Cell at a Time. Non-Coding RNA.

[185] Herbst E., Mandel-Gutfreund Y., Yakhini Z., Biran H. (2025). Inferring Single-Cell and Spatial microRNA Activity from Transcriptomics Data. Commun. Biol..

[186] Wang Y., Aivalioti E., Stamatelopoulos K., Zervas G., Mortensen M.B., Zeller M., Liberale L., Di Vece D., Schweiger V., Camici G.G. (2025). Machine Learning in Cardiovascular Risk Assessment: Towards a Precision Medicine Approach. Eur. J. Clin. Investig..

[187] Nordestgaard L.T., Wolford B.N., de Gonzalo-Calvo D., Sopić M., Devaux Y., Matic L., Wettinger S.B., Schmid J.A., Amigó N., Masana L. (2025). Multiomics in Atherosclerotic Cardiovascular Disease. Atherosclerosis.

