# Supplementary material for: MicroRNAs in Cardiovascular Diseases: Molecular Networks of Cellular Homeostasis, Inflammation, and Pathological Remodeling
Source: Int J Mol Sci. 2026 Apr 17;27(8):3582. doi: 10.3390/ijms27083582 (PMC13116442; doi:10.3390/ijms27083582)
Supplement: Supplementary file 1 [file ijms-27-03582-s001.zip › ijms-4255321-supplementary.pdf]

Table S1. Representative studies included in the review and qualitative appraisal of evidence strength.

Cardiovascular context	Study type / model	Main miRNA-related finding	Validation / replication	Strength of evidence	Key limitations	References
Endothelial homeostasis / angiogenesis	Experimental endothelial and vascular models	miR-126 is linked to vascular integrity and angiogenic competence	Supported by independent early endothelial studies	High	Mainly preclinical; limited direct clinical outcome data	[9]
VSMC phenotypic regulation	Mouse + human vascular correlation	miR-143/145 regulates VSMC differentiation and phenotype switching	Supported by animal and human vascular evidence	High	Not a clinical intervention study	[18]
Post-MI fibrosis	Experimental MI/fibrosis models	miR-29 downregulation is linked to profibrotic remodeling	Repeatedly supported in later fibrosis literature	High	Mainly preclinical	[31]
Cardiac fibrosis / fibroblast activation	Experimental cardiac fibrosis models	miR-21 promotes fibroblast activation and fibrotic signaling	Foundational mechanistic study with broad later support	High	Limited direct clinical validation	[43]

Arrhythmogenesis	Experimental cardiac electrophysiology models	miR-1 regulates conduction-related targets and arrhythmogenic susceptibility	Widely echoed in later arrhythmia literature	High	Mostly experimental; limited direct human validation	[26]
Atherosclerosis / lipid handling	Mouse interventional model	Anti-miR-33 improves cholesterol efflux and reverse cholesterol transport	Strong preclinical intervention evidence	Moderate	Non-human model; translation to patients remains indirect	[82]
AMI biomarker development	Human plasma cohort	Circulating miRNAs rise early after AMI and may support diagnosis	Foundational human biomarker study	Moderate	Early biomarker work; later studies highlight pre-analytical variability	[97]
Post-MI ventricular arrhythmogenic remodeling	Experimental post-MI inflammatory model	miR-155 links macrophage-driven inflammation with sympathetic neural remodeling and ventricular arrhythmia susceptibility after MI	Mechanistically coherent with other inflammatory arrhythmia studies	Moderate	Preclinical; limited direct human validation	[102]

Heart failure biomarkers (HFrEF/HFpEF)	Meta-analysis / individual patient data	Composite miRNA panels may provide prognostic value across HF phenotypes	Broader patient-based evidence than single-cohort studies	High	Dependent on heterogeneity of included studies	[107]
HFpEF / therapeutic targeting	Experimental HFpEF model	Anti-miR-92a improves vascular gene expression and diastolic phenotype	Strong preclinical signal for phenotype-specific therapy	Moderate	Preclinical only; no clinical HFpEF trial validation yet	[109]
Heart failure therapy / RNA therapeutics	Early-phase human clinical trial	Anti-miR-132 showed translational feasibility and safety signals	Human interventional data	High	Early-phase study; efficacy conclusions remain preliminary	[157]
Atrial fibrillation / atrial fibrosis	Human left atrial expression study	miR-146b-5p is associated with atrial fibrosis-related remodeling	Human tissue-based evidence	Moderate	Limited cohort size; disease-specific context	[116]

Abbreviations: AMI, acute myocardial infarction; HF, heart failure; HFpEF, heart failure with preserved ejection fraction; HFrEF, heart failure with reduced ejection fraction; miRNA, microRNA; post-MI, post-myocardial infarction; VSMC, vascular smooth muscle cell; Cx43, connexin 43.
